# Exposure to Perflouroalkyl acids and foetal and maternal thyroid status: a review

**DOI:** 10.1186/s12940-020-00647-1

**Published:** 2020-10-13

**Authors:** Sophie A. H. Boesen, Manhai Long, Maria Wielsøe, Vicente Mustieles, Mariana F. Fernandez, Eva C. Bonefeld-Jørgensen

**Affiliations:** 1 Centre for Arctic Health and Molecular Epidemiology, Department of Public Health, Aarhus University, Aarhus, Denmark; 2 School of Medicine, Center of Biomedical Research, University of Granada, Granada, Spain; 3 Consortium for Biomedical Research in Epidemiology & Public Health (CIBERESP), Madrid, Spain; 4 Greenland Centre for Health Research, University of Greenland, Nuuk, Greenland

**Keywords:** Perfluorinated-alkyl-acids, PFAAs, Thyroid hormones, TSH, Maternal, Infant, Mother-child-health, Human biomonitoring, HBM4EU

## Abstract

**Background:**

Exposure to perfluorinated-alkyl-acids (PFAAs) is ubiquitous. PFAAs are hormone-disrupting compounds that are strongly suspected to affect mother-child-health such as fetal growth. Thyroid disruption is a plausible mechanism of action. We aim to summarize the epidemiological evidence for the relation between prenatal and postnatal exposure to PFAAs and disruption of thyroid homeostasis in mothers and/or infants.

**Method:**

Fifteen original publications on PFAAs concentrations and thyroid hormones (TH) in pregnant women and/or infants were found upon a literature search in the PubMed database. Information on exposure to seven PFAAs congeners [Perfluorooctane sulfonate (PFOS), Perfluorooctanoate (PFOA), Perfluorohexane sulfonate (PFHxS), Perfluorononanoic acid (PFNA), Perfluorodecanoic acid (PFDA), Perfluoroundecanoic acid (PFUnA), and Perfluorododecanoic acid (PFDoA)] and thyroid stimulating hormone (TSH), free and total thyroxine (FT4 and TT4), free and total triiodothyronine (FT3 and TT3), T3RU (Free triiodothyronine resin uptake) and FT4-index (FT4I) levels were recorded. We evaluated sampling of maternal TH by trimester, and infant TH by sex stratification. Reported associations between mother or infant PFAAs and TH were not uniformly assessed in the selected studies.

**Results:**

Ten out of the fifteen studies examined maternal PFAAs concentration and TSH level. Seven studies showed significant associations between TSH and exposure to six PFAAs congeners, most of them were positive. Maternal T4 and T3 were investigated in nine studies and five studies found inverse associations between exposure to six PFAAs congeners and TH (TT3, TT4, FT3, FT4 and FT4I) levels.

Eight of the fifteen studies investigated PFAAs concentrations and infant TSH. Infant TSH level was significantly affected in four studies, positively in three studies. Nine studies investigated infant T4 and T3 and seven studies found significant associations with PFAAs exposure. However, both inverse and positive significant associations with infant TH were found eliciting no clear direction.

**Conclusion:**

Results indicate a mainly positive relationship between maternal PFAAs concentrations and TSH levels, and suggestion of an inverse association with T4 and/or T3 levels. Associations of infant TH with PFAAs concentration were less consistent.

## Background

Increasing evidence suggests that in utero exposure to perfluorinated-alkyl-acids (PFAAs), a subgroup of persistent organic pollutants (POPs), have adverse effects on fetal growth and development [1]. Exposure to PFAAs has also been shown to affect the thyroid function [2–4], decrease vaccination immune response in children [5, 6], increase the risk of breast cancer [7], and the development of metabolic syndrome, type 2 diabetes [8], lipoedema and cardiovascular disease [1, 9, 10].

PFAAs are detected globally in human populations and wildlife [11–13]. Humans are mainly exposed to PFAAs through food items and drinking water, but also by inhalation and dermal exposure [14]. The mean half-life of serum elimination in humans is 5.4 years for perfluorooctane sulfonate (PFOS), 3.8 years for perfluorooctanoate (PFOA) and 8.5 years for perfluorohexane sulfonate (PFHxS) [15]. Since 2000, several western countries have made restrictions on the PFAAs production [16], but presently only PFOS and PFOA are regulated by the Stockholm Convention on Persistent Organic Pollutants [17]. Thus, human serum concentrations, especially for the regulated PFOS and to some degree PFOA, have decreased during the last decade [18, 19].

PFAAs enter the foetal circulation through placenta transfer and postnatally the infant is exposed by breastfeeding [20]. Thus, the foetus is exposed to PFAAs at highly vulnerable developmental stages [21]. Disruption of hormonal pathways involving the hypothalamic-pituitary-thyroid (HPT) and hypothalamic-pituitary-gonadal (HPG) axes has been hypothesized as a mechanism of action (MoA) and mediating steps between the effects of PFAAs on foetal growth [22]. Maternal abnormal thyroid function during pregnancy might affect foetal growth and neural development [23–28]. The foetus’ thyroid hormone (TH) production does not suffice until midgestation but rely on maternal supply mainly during the 1st trimester, implying a high demand on the maternal HPT axis [23].

Maternal thyroxine (T4) and triiodothyronine (T3) hormones crosses the placenta, but not the maternal thyroid stimulating hormone (TSH) [29, 30]. In the pregnant woman, the TH production undergoes dynamic and physiological changes: TSH decreases during the 1st trimester and stabilizes in the 2nd and 3rd trimester [31]. The European Thyroid Association guidelines recommend a TSH upper reference limit of 2.5 mIU/L in the 1st trimester and 3.0 mU/ L in the second and third trimesters [32], though it is discussed whether the limits are too narrow [31]. In the absence of pre-existing thyroid disease, the American Thyroid Association guideline recommends upper limit of 4mIU/L in early pregnancy week 7–12 and gradually during 2nd and 3rd trimesters a return to non-pregnancy range [33]. High human corionic gonadotropin possess a thyrotrophic effect and elicit a physiological increase in levels of T4 and T3. High oestrogen levels in pregnancy increase circulating levels of thyroxin binding globulin (TBG), and may lead to an increase in the total level of T4 and T3 [34, 35]. The thyroid binding capacity is facilitated by several plasma proteins such as TBG, transthyretin (TTR) and albumin [36]. Thyroid peroxidase (TPO) is a key enzyme of TH biosynthesis in the thyroid gland [37]. Thyroid peroxidase-antibodies (TPOAb) may potentially be an important covariate. TPOAb production during pregnancy might be indicative of thyroid stress especially when levels are above a cut-off value [26].

This review will focus on exposure to PFAAs and thyroid hormone homeostasis in the mother and infant. Effect estimates were evaluated with the objective to understand whether PFAAs exposure is related to hypothyroidism (low free T4, high TSH) or hyperthyroidism (high free T4, low TSH).

## Method

The PubMed database was searched, using Medical Subject Headings (MESH) terms and specific keywords, to locate relevant publications, following a specific strategy (Table 1). Last literature search was conducted on the 21th January 2020. Citations from selected publications were also investigated. This report was restricted to epidemiological studies with a sample size > 100 participants. All selected studies are summarized in Tables 2, 3, 4, 5, 6, 7, 8, 9, and 10.

**Table 1:** Literature search strategy

Database	MESH and non-MESH terms		Hits
Pubmed, sorted by ”Best Match”		(Perfluorinated OR Perfluorinated acid OR perfluorooctane sulfonic acid OR perfluorooctanoic acid OR fluorocarbons OR polyfluorinated OR polyfluoroalkyl OR perfluoroalkyl Perfluorochemicals OR PFOS OR PFOA OR PFNA OR PFDA OR PFHxS OR PFUnA OR PFOSA OR PFDA)	17,054
	AND	(Thyroid OR Thyroid hormones OR thyroxine OR triiodothyronine OR thyroid-stimulating hormone OR TSH OR T4 OR T3)	223
	AND	(Pregnancy OR maternal OR mother OR newborn OR infant OR cord)	59
Studies full filling inclusion criteria	Participants	Pregnant women and/or infants with measurements of PFAAs exposure and thyroid hormones.	20
	Number	> 100 participants.	15

### Data extraction

Following data was extracted to supply general information and study characteristics (Table 2): Study design and population, year of enrollment, number of participants, exclusion criteria, biological matrix for exposure assessment, pregnancy trimester of exposure assessment and TH outcome measurement, PFAA compounds examined above the limits of detection/quantification (LOD/LOQ), outcome estimates and confounders.

Tables 3 and 4 give the information on exposure, including trimester of PFAAs measurement, participants, median concentration (ng/mL) and % < LOD/LOQ. Tables 5 and 6 give the maternal and infant TH levels, respectively. Tables 7, 8, 9 and 10 show the original data on thyroid hormone outcome upon PFAAs exposure.

## Results

### Study populations

We found 15 original publications that fulfilled the inclusion criteria [38–52] (Table 2). Study populations varied from 118 to 1366 participants [40, 51], conducted in Asia (*n* = 6), Europe (*n* = 6) and Northern America (*n* = 3). Study designs were cross sectional and/or cohort studies. Most studies adjusted for maternal age and/or parity (Table 2).

**Table 2:** Presentation of included studies

[Reference]	Author Country Year	Study design	Year of enrolment	Number of participants	Study population	Exclusion criteria	Biological material matrix for Exposure assessment	Time point in pregnancy of PFAAs exposure and TH outcome collection	Contaminants examined above> LOD/LOQ	Outcome	Adjusted for
[38]	Kato Japan 2016	Cohort study	2002–2005	*N* = 392	Participants from the Hokkaido Study on the Environment and Children’s Health cohort.	Treatment for thyroid disease and incomplete dataset.	Maternal serum	Maternal PFAAs measured 1 time during 2nd, 3rd trimester or within 5 days after delivery Maternal TH: Median week: 11.1 (1st Trimester) Infant TH: 4–7 days postpartum (heelprick)	PFOS PFOA	Maternal and infant TSH and FT4	*Maternal PFAAs and maternal TH*: maternal age, BMI, parity, educational level, thyroid antibody, intake of seaweed, blood sampling period before/after delivery for PFOS and PFOA and gestational week for TH sampling *Maternal PFAAs and infant TH*: Maternal factors (maternal age at delivery, parity, thyroid antibody, blood sampling period before/after delivery for PFOS and PFOA and values of TSH and FT4); and infant factors (gestational weeks at birth, low birth weight\2500 g and mode of delivery)
[39]	Preston USA 2018	Cross sectional/cohort study	1999–2002	*N*(maternal) = 732 *N*(infant) =480	Participants recruited from Project Viva Birth Cohort.	Prior or current diagnosis of thyroid disease and/or current thyroid medication use.	Maternal plasma	Maternal PFAAs and TH: collected before 14th week. (1st Trimester) Infant TH: 3 days postpartum (heel prick)	PFHxS PFOS PFOA PFNA EtFOSAA MeFOSAA	Maternal TT4, FT4I and TSH. TPOAb investigated. Infant TT4	*Maternal PFAAs and maternal TH*: age, parity, gestational week at blood draw, ethnicity, fish intake and smoking *Maternal PFAAs and infant TH*: maternal factors and infant sex, gestational age, delivery type.
[40]	Inoue Denmark 2019	Cross sectional	1996–2002	*N* = 1366	Participants from the Danish National Birth Cohort (DNBC)	Absence of PFAAs or TH measurement	Maternal plasma	Maternal PFAAs and TH: Week 5–19 (Median week 8) (1st Trimester)	PFHxS PFHpS PFOS PFOA PFNA PFDA	Maternal TSH and FT4	Maternal age at delivery, parity, socio-occupational status, maternal pre-pregnancy BMI, gestational week of blood sampling measure, maternal smoking during pregnancy, child’s year of birth
[41]	Wang Norway 2013	Cross sectional	2003–2004	*N* = 903	Participants from the Norwegian Mother and Child Cohort Study (MoBa).	Thyroid abnormality or medication.	Maternal plasma	Maternal PFAAs and TH: Week: 17–18 (2nd Trimester)	PFHxS PFOS PFOA PFNA PFDA PFUnA PFHpS	Maternal TSH	Maternal age, gestational age at blood draw, high density lipid concentrations, total seafood intake, parity and inter-pregnancy interval.
[42]	Webster Canada 2014	Cohort study	2007–2008	*N* = 152	Participants recruited from the Chemicals Health and Pregnancy study (CHirP) study.	< 15 weeks pregnant, > 19 years old, smokers, multiple pregnancies, conceived with assistance, thyroid or endocrine condition, consummation of medication affecting TH levels.	Maternal serum	Maternal PFAAs: Week 15 (2nd trimester) Maternal TH: Measured twice at week 15 and week 18 (2nd trimester)	PFHxS PFOS PFOA PFNA	Maternal FT4, TT4 and TSH. TPOAb investigated.	Week of gestation and TPOAb status.
[43]	Berg Norway 2015	Cohort study	2007–2009	*N* = 391	Women from the Northern Norway Mother-and-Child Contaminant Cohort Study (MISA).	Incomplete dataset, thyroid disease and twin pregnancies.	Maternal serum	Maternal PFAAs: Median gestational week 18 (range: 10–34) (2nd trimester). Maternal TH were measured 3 times at 3 visits: 1st visit: 2nd trimester (week 13–28) 2nd visit: 3 days postpartum 3rd visit: 6 weeks postpartum	PFHxS PFOS PFOA PFNA PFDA PFUnA PFHpS	Maternal TSH, T3, T4, FT3, FT4	Parity, age, BMI and thyroxin binding capacity
[44]	Wang Taiwan 2014	Cohort study	2000–2001	*N* = 285	Participants recruited from the Taiwan Maternal and Infant Cohort Study.	Incomplete dataset, thyroid disease or medication.	Maternal serum	Maternal PFAAs and TH: Week 28–40 (3rd trimester) Infant TH: At delivery (cord blood serum)	PFHxS PFOS PFOA PFNA PFDA PFUnA PFDoA	Maternal and infant FT4, TT4, TT3 and TSH	*Maternal PFAAs and maternal TH:* Maternal age, maternal education levels and maternal previous live births. *Maternal PFAAs and cord TH:* Maternal age, maternal education levels, maternal previous children, neonatal sex and neonatal type of delivery
[45]	Reardon Canada 2019	Prospective cohort study	2009–2012	*N* = 494	The Alberta pregnancy outcomes and nutrion (Apron) cohort.	Fertility treatment, smoking during pregnancy and missing demographic data.	Maternal plasma (86%)/sera (14%)	Maternal PFAAs was measured at timepoint 2. TH was sampled at all timepoints. Timepoint 1: < 13 week, *n* = 167 Timepoint 2: 14–27 week, *n* = 487 Timepoint 3: 27–40 week, *n* = 465 Timepoint 4: 3 months postpartum, *n* = 479	PFHxS PFOS PFOA PFNA PFDA PFUnA PFDoA PFHpA Isomers of PFOS(*n* = 6)	Maternal TSH, FT4 and FT3 TPOAb investigated	Maternal age, education, ethnicity, household income, parity, medical conditions, smoking, alcohol, recreational drug use and hypothyroid medications.
[46]	Yang China 2016	Cross sectional	2013	*N* = 157	Healthy local resident women with a singleton live-born child without congenital anomalies were randomly recruited.	Medical histories of thyroid diseases or any other organic diseases.	Maternal serum and cord blood serum	Maternal PFAAs and TH: 1–2 days before delivery (3rd Trimester) Infant TH: At delivery (cord blood serum)	PFHxS PFOS PFOA PFNA PFDA PFUnA PFDoA 6:2 FTS NMeFOSAA	Maternal and infant FT3, FT4, T3, T4 and TSH	*Maternal PFAAs and maternal TH:* Age, BMI, monthly income and type of delivery *Maternal PFAAs and cord TH:* As maternal TH and maternal previous live births
[47]	Xiao Faroe Island 2019	Prospective cohort study	1994–1995	*N* = 172		Children born before 36th week of gestation, birth weight < 2500 g, any congenital neurological diseases	Maternal serum	Maternal PFAAs and TH: Week 34 (3rd Trimester) Infant TH: At delivery (cord blood serum)	PFHxS PFHpS PFOS PFHpA PFOA PFNA PFDA PFUnA PFDoA NEtFOSAA NMeFOSAA FOSA	Maternal TSH, FT4, FT3, T3RU, FT4I Infant TSH, FT4, FT3, T3RU, FT4I	Infant sex, gestational age in weeks, maternal education, maternal pre-pregnancy BMI, parity, smoking status during pregnancy, alcohol consumption during pregnancy, maternal hair-mercury, maternal serum sumPCB138, 153, 180
[48]	Dufour Belgium 2018	Cross sectional	2013–2016	*N* = 214	Women presenting for delivery at the obstetric service of the University Hospital of Liege (Belgium).	Insufficient serum volume available (< 0.5 mL), absence of TSH level record, congenital hypothyroidism diagnosed for the new born (TSH > 20 mUI/L).	Cord blood serum	Infant PFAAs exposure: At delivery (cord blood serum) Infant TH: 3 days postpartum (heel prick)	PFHxS PFOS PFOA PFNA PFDA PFUnA PFHpA	Infant TSH	Parity, gender and gestational age.
[49]	Shah-Kulkarni Korea 2016	Cross sectional study	2006–2010	*N* = 279	Participants from the Ewha Birth & Growth Retrospective Cohort (EBGRC).	Pregnancy complications (hypertension and diabetes) and missing information for gestational age and gender.	Cord blood serum	Infant PFAA exposure and TH outcome: At delivery (cord blood serum)	PFHxS PFOS PFOA PFNA PFDA PFUnA PFDoA PFTeDA PFTrDA PFPeA	Infant T3, T4 and TSH	Age, education, pre-pregnancy body mass index, alcohol intake history, parity, gender and gestational age
[50]	De Cock Netherland 2014	Cross sectional study	2011–2013	*N* = 148	Participants from the Linking EDCs in maternal nutrion to child health (LINC) study.	Twin pregnancies and major congenital anomalies.	Cord blood plasma	Infant PFAAs exposure: At delivery (cord blood plasma) Infant TH outcome: 4–7 days postpartum (heel prick)	PFOS PFOA	Infant TT4	Maternal thyroid gland problems, use of thyroid medication, parity, smoking, alcohol, BMI and age at birth. Infant birth weight, gestational weight gain and gestational age.
[51]	Tsai Taiwan 2017	Cross sectional	2004–2005	*N* = 118	The Taiwan Birth Panel Study (TBPS).	Absence of PFAAs or TH measurement	Cord blood plasma	Infant PFAA exposure and TH outcome: At delivery (cord blood plasma)	PFOA PFOS PFNA PFUnA	Infant TSH and T4	Age, BMI, parity, gestational week at blood draw, educational level, infant sex, gestational age, delivery type
[52]	Aimuzi China 2019	Cross sectional	2012–2013	*N* = 568	The Shanghai Obesity and allergy Cohort Study	Thyroid disease, lack of information on thyroid function, infrapartum cesearean delivery and preterm birth.	Cord blood plasma	Infant PFAA exposure At delivery (cord blood plasma) Infant TH outcome: At delivery (cord blood serum)	PFBS PFHxS PFOS PFHpA PFOA PFNA PFDA PFUnA PFDoA PFOSA	Infant FT4, FT3 and TSH	Maternal age, infant sex, maternal parity (nulliparous or parous), gestational age at birth, maternal pre-pregnancy BMI and maternal fish intake during pregnancy.

TH (thyroid hormone), TT3 (total triiodothyronine), FT3 (free triiodothyronine), TT4 (total thyroxine), FT4 (free thyroxine), FT4I (free thyroxine index); T3RU (Free triiodothyronine resin uptake)

*PFOS* Perfluorooctane sulfonate, *PFOA* Perfluorooctanoate, *PFHxS* Perfluorohexane sulfonate, *PFHpS* perfluoroheptane sulfonate, *PFHpA* Perfluoroheptanoic acid, *PFNA* Perfluorononanoic acid, *PFDA* Perfluorodecanoic acid, *PFUnA* Perfluoroundecanoic acid, *PFDoA* Perfluorododecanoic acid, *EtFOSAA* 2-(N-ethyl-perfluorooctane sulfonamido)acetate, *MeFOSAA* 2-(N-methyl-per-fluorooctanesulfonamido)acetate, *6:2 FTS* 6:2 fluorotelomer sulfonates, *NMeFOSAA* N-methyl perfluorooctane sulfonamidoacetate, *NEtFOSAA* N-ethyl perfluorooctane sulfonamidoacetate, *FOSA* Perfluorooctane sulphonamide, *PFTeDA* Perfluoro tetradecanoic acid, *PFTrDA* Perfluorotridecanoic acid, *PFPeA* perfluoro-npentanoic acid, *PFBS* perfluorobutane sulfonate, *PFOSA* perfluorooctane sulfonamide, *LOD/LOQ* limit of detection/quantification

### PFAAs exposure measurement

Tables 3 and 4 show the PFAAs level in mothers and infants, respectively. PFAAs exposure was assessed in maternal plasma or serum (10 studies, Tables 2 and 3) collected in 1st trimester (*n* = 2) [39, 40], 2nd trimester (*n* = 5) [38, 41–43, 45] or 3rd trimester (*n* = 4) [38, 44, 46, 47]. One study measured the maternal PFAAs level between 24 and 41 weeks of gestational age (2nd, 3rd trimester) or within 5 days after delivery [38]. PFAAs were also assessed in cord blood serum / plasma (*n* = 6) [46, 48–52] (Tables 2 and 4).

**Table 3:** Concentrations of maternal PFAAs

				PFOS			PFOA			Other PFAAs			
Reference	Author Country Year	Time point of exposure collection	Number	Median (ng/mL)	LOD/LOQ (ng/mL)	% < LOD/LOQ	Median (ng/mL)	LOD/LOQ (ng/mL)	% < LOD/LOQ	PFAAs	Median (ng/mL)	LOD/LOQ (ng/mL)	% < LOD/LOQ
[39]	Preston USA 2018	<14th week. (1st trimester)	732	24.0	0.2	NR	5.6	0.1	NR	PFHxS	0.6	0.1	NR
										PFNA	2.4	0.1	
										EtFOSAA	1.1	0.1	
										MeFOSAA	1.8	0.1	
[40]	Inoue Denmark 2019	Median week 8 (1st trimester)	1366	29.5	0.28	0%	4.52	0.20	0%	PFHxS^a^	1.11	0.08	0.2%
										PFHpS^a^	0.37	0.11	1%
										PFNA^a^	0.45	0.27	6%
										PFDA^a^	0.17	0.09	5%
[41]	Wang Norway 2013	Week: 17–18 (2nd trimester)	903	12.81	0.05	0%	2.15	0.05	0%	PFHxS	0.6	0.05	1%
										PFNA	0.39	0.05	1%
										PFUnA	0.22	0.05	6%
										PFHpS	0.13	0.05	12%
										PFDA	0.04	0.05	31%
[42]	Webster Canada 2014	Median week: 15–18 (2nd trimester)	152	4.8	0.5	0%	1.7	0.5	1.3%	PFHxS	1.0	0.5	15.7%
										PFNA	0.6	0.5	31.6%
[43]	Berg Norway 2015	Median week 18 (2nd trimester)	391	8.03	0.31	NR	1.53	0.07	NR	PFHxS	0.44	0.06	NR
										PFHpS	0.10	0.06	
										PFNA	0.56	0.04	
										PFUnA	0.26	0.02	
										PFDA	0.23	0.03	
[45]	Reardon Canada 2019	Week 14–27 (2nd trimester)	494	4.77	0.01–0.12	0%	2.11	0.01–0.37	0%	PFHxS	1.03	0.03	0%
										PFNA	0.69	0.06	1%
										PFDA	0.26	0.04	0.2%
										PFUnA	0.06	0.03	11.5%
										PFDoA	0.06	0.02	44.5%
										PFHpA	0.08	0.02	33.3%
[38]	Kato Japan 2016	2nd -3rd Trimester	392	5.2	0.5	NR	1.2	0.5	NR	–	–	–	–
[44]	Wang Taiwan 2014	Week 28–40 (3rd trimester)	285	12.73	0.07–0.45	0%	2.39	0.07–0.45	13.0%	PFHxS	0.81		22%
										PFNA	1.51		4%
										PFUnA	3.26	0.07–0.45	9%
										PFDeA	0.46		29%
										PFDoA	0.36		18%
[46]	Yang China 2016	1–2 days before delivery (3rd trimester)	157	4.41	0.021	0%	1.64	0.024	0%	PFHxS	0.50	0.012	0%
										PFNA	0.46	0.013	0%
										PFDA	0.37	0.024	0%
										PFUnA	0.40	0.033	1%
										PFDoA	0.041	0.029	32%
										6:2 FTS	0.041	3	18%
										MeFOSAA	0.003	0.3	26%
[47]	Xiao Faroe Island 2019	Week 34 (3rd Trimester)	172	20.86^b^	0.03	0%	2.37^b^	0.03	0%	PFHxS	0.55^b^	0.03	0%
										PFHpS	0.35^b^	0.03	7%
										PFHpA	0.03^b^	0.03	53%
										PFNA	0.60^b^	0.03	0%
										PFDA	0.30^b^	0.03	0%
										PFUnA	0.47^b^	0.03	0%
										PFDoA	0.03^b^	0.03	68%
										NEtFOSAA	0.65^b^	0.03	0%
										NMeFOSAA	0.18^b^	0.03	0%
										FOSA	0.04^b^	0.03	63%

% < LOD/LOQ: percentage below limit of detection/quantification, NR: information not reported, −: no other PFAAs examined, ^a^ only measured in a sub-group of the study population (*n* = 1178), ^b^ Geometric mean

*PFOS* Perfluorooctane sulfonate, *PFOA* Perfluorooctanoate, *PFHxS* Perfluorohexane sulfonate, *PFHpS* Perfluoroheptane sulfonate, *PFHpA* Perfluoroheptanoic acid, *PFNA* Perfluorononanoic acid, *PFDA* Perfluorodecanoic acid, *PFUnA* Perfluoroundecanoic acid, *PFDoA* Perfluorodo-decanoic acid, *EtFOSAA* 2-(N-ethyl-perfluorooctane sulfonamido)acetate, *MeFOSAA* 2-(N-methyl-per-fluorooctanesulfonamido)acetate, *6:2 FTS* 6:2 fluorotelomer sulfonates, *NMeFOSAA* N-methyl perfluorooctane sulfonamidoacetate, *NEtFOSAA* N-ethyl perfluorooctane sulfonamidoacetate, *FOSA* Perfluorooctane sulphonamide

**Table 4:** Concentrations of infant PFAAs

				PFOS			PFOA			Other PFAAs			
Reference	Author Country Year	Time point of exposure collection	Number	Median (ng/mL)	LOD/LOQ (ng/mL)	% < LOD/LOQ	Median (ng/mL)	LOD/LOQ (ng/mL)	% < LOD/LOQ	PFAAS congener	Median (ng/mL)	LOD/LOQ (ng/mL)	% < LOD/LOQ
[46]^a^	Yang China 2016	Cord blood serum at delivery	157	1.18	0.021	0%	1.15	0.024	0%	PFHxS	0.18	0.012	0%
										PFNA	0.20	0.013	0%
										PFDA	0.10	0.024	0%
										PFUnA	0.12	0.033	4%
										PFDoA	0.021	0.029	67%
										6:2 FTS	0.025	3	10%
										NMeFOSAA	0.0006	0.3	34%
[48]	Dufour Belgium 2018	Cord blood serum at delivery	214	0.73	0.50	29%	0.68	0.25	5.7%	PFHxS	0.16	0.15	45.6%
										PFHpA	<LOQ	0.05	92.7%
										PFNA	0.15	0.10	24.9%
										PFDA	<LOQ	0.15	91.2%
										PFUnA	<LOQ	0.10	95.9%
[49]	Shah-Kulkarni Korea 2016	Cord blood serum at delivery	279	0.66	0.11/0.33	26%	0.91	0.08/0.24	7%	PFHxS	0.38	0.16/0.48	69%
										PFUnA	0.26	0.08/0.24	44%
										PFPeA	0.2	0.08/0.24	44%
										PFNA	0.2	0.05/0.15	36%
										PFDA	0.1	0.06/0.18	87%
										PFDoA	0.08	0.05/0.15	97%
										PFTeDA	0.06	0.09/0.27	100%
										PFTrDA	0.39	0.15/0.45	57%
[50]^b^	De Cock Netherland 2014	Cord blood plasma at delivery	148	1.6	5	0%	0.89	7	0%	–	–	–	–
[51]	Tsai Taiwan 2017	Cord blood plasma at delivery	118	5.11	0.066/0.22	0%	2.55	1.23/1.58	18%	PFNA	3.57	2.4/3.1	68.5%
										PFUnA	3.57	0.67/0.84	85.5%
[52]	Aimuzi China 2019	Cord blood plasma at delivery	568	2.51	0.009–0.12	0%	7.57	0.009–0.12	0%	PFHxS	0.18	0.009–0.12	0%
										PFNA	0.66		0%
										PFDA	0.41		0.4%
										PFUnA	0.40		0.2%
										PFDoA	0.11		6.9%
										PFBS	0.05		1.6%
										PFOSA	<LOD		19.5%
										PFHpA	<LOD		54%

% < LOD/LOQ: percentage below limit of detection/quantification, −: no other PFAAs examined

*PFOS* Perfluorooctane sulfonate, *PFOA* Perfluorooctanoate, *PFHxS* Perfluorohexane sulfonate, *PFHpA* Perfluoroheptanoic acid, *PFNA* Perfluorononanoic acid, *PFDA* Perfluorodecanoic acid, *PFUnA* Perfluoroundecanoic acid, *PFDoA* Perfluorododecanoic acid, *6:2 FTS* 6:2 fluorotelomer sulfonates, *NMeFOSAA* N-methyl perfluorooctane sulfonamidoacetate, *PFTeDA* Perfluoro tetradecanoic acid, *PFTrDA* Perfluorotridecanoic acid, *PFBS* Perfluorobutane sulfonate, *PFOSA* perfluorooctane sulfonamide, ^a^Studies did not use foetal PFAAs concentrations in the analysis given in this review. They only used maternal PFAAs exposure

^b^: The PFAAs levels were given as ng/L in the original publication, and changed to ng/ml for comparison with the other studies

In all studies, PFAAs were measured using liquid chromatography mass-spectrometry techniques. We have in this review focused on seven PFAAs congeners ((n) studies): PFOS (*n* = 15), PFOA (*n* = 15), PFHxS (*n* = 12), PFNA (*n* = 13), PFDA (*n* = 10), PFUnA (*n* = 10), and PFDoA (*n* = 6), although the studies also examined other congeners [PFHpS, EtFOSAA, MeFOSAA, 6:2 FTS, NEtFOSAA, NMeFOSAA, PFPeA, PFHpA, PFTrDA, PFTeDA and PFOS isomers], which were not included in this review. The range of maternal PFOS median concentration was 4.4 ng/mL [46] to 29.5 ng/mL [40] (Table 3). Among infants, the range of the PFOS concentration was 0.66 ng/mL [49] to 5.11 ng/mL [51] in umbilical cord blood serum/plasma (UCB) samples (Table 4).

### Thyroid hormone assessment

The measurement of thyroid hormones varied between the studies, showing mainly TSH, free T4 (FT4), total T4 (TT4), free T3 (FT3) and total T3 (TT3) levels or variations hereof, i.e. FT4 index (FT4I), calculated from TT4 and T3-resin uptake (FT4I = TT4 × T3 resin uptake) [39, 47].

Maternal TH were measured during the 1st trimester (*n* = 3) [38–40], 2nd trimester (*n* = 2) [41, 42], 3rd trimester (*n* = 3) [44, 46, 47] or at multiple time points (*n* = 2) [43, 45]. Infant TH were assessed postpartum in cord blood serum/plasma (*n* = 6) [44, 46, 47, 49, 51, 52] or infant serum obtained at heel stick up to 7 days postpartum (*n* = 4) [38, 39, 48, 50] (Table 2).

Thus, maternal PFAAs exposure and maternal TH levels were predominantly measured simultaneously with exception of Kato et al. who measured maternal TH in the 1st trimester, while PFAAs exposure was measured after 1st trimester [38] (Table 2).

Tables 5 and 6 shows the maternal and infant TH levels, respectively. The maternal TSH median levels were in general lower than the upper reference limit with no obvious trimester differences. In general similar TT3 and FT3 levels were observed among the reviewed studies, as expected with lower FT3 (pmol/L) levels than the TT3 (nmol/L) levels. Also the FT4 and TT4 levels were in overall similar among the studies with significantly higher TT4 levels (Table 5). Four studies [39, 42, 43, 45] also determined the maternal TPOAb finding positive levels.

**Table 5:** Maternal TH levels

Reference	Author Country Year	Time point of TH measurement		TSH			T3			T4			TPOAb
			Number		Median	Range		Median	Range		Median	Range	
*Normal range in non-pregnant women#*						0.300–4.5 IU/L			1.10–2.50 nmol/L			60–140 nmol/L	–
*Normal range in pregnant women* American Thyroid Association				1st Trimester	Upper reference limit of 4.0 mU/L								
				2nd-3th Trimester	Gradually return to non-pregnancy range								
European Thyroid Association				1st Trimester	Upper reference limit of 2.5 mU/L								
				2nd-3th Trimester	Upper reference limit of 3.0 mU/L								
*Studies included in review*													
[38]	Kato Japan 2016	Median week: 11.1	392	TSH (mIU/L)*	1.0	LOD-4.1^a^				FT4 (pmol/L)*^, d^	12.7	7.9–21.56 ^a^	
[39]	Preston USA 2018	<14th week. (1st trimester)	732	TSH (mIU/mL) ^e^	1.2	< 0.01–21.4				TT4 (nmol/L)*	127.4	50.2–314.1	Positive (> 2 U/mL): 14%
										FT4Index	2.1	1.3–6.0	
[40]	Inoue Denmark 2019	Week 5–19 (Median week 8) (1st Trimester)	1366	TSH (mIU/L)	1.13^b^	1.08–1.19 ^a^				FT4 (pmol/L)	14.2^b^	14.1–14.3 ^a^	
[41]	Wang Norway 2013	Week 17–18 (2nd trimester)	903	TSH (mIU/L)	3.52	Max: 18.64							
[42]	Webster Canada 2014	Week 15 (2nd trimester)	150	TSH (mIU/L)	1.3	0.0–3.3				TT4 (nmol/L)	126.4	77.4–200.1	High (≥ 9 IU/mL): 9%
										FT4 (pmol/L)	9.5	7.3–12.9	
		Week 18 (2nd trimester)		TSH (mIU/L)	1.3	0.2–4.0				TT4 (nmol/L)	119.1	65–7-175.7	
										FT4 (pmol/L)	8.5	5.8–11.1	
[43]	Berg Norway 2015	2nd trimester (week 13–28)	375	TSH (mIU/L)	1.55	0.06–10.2	TT3 (nmol/L)	2.71	1.47–4.75	TT4 (nmol/L)	145	92.00–215	Positive (> 34 IU/L): 6%
							FT3 (pmol/L)	4.59	2.99–7.08	FT4 (pmol/L)	13.0	9.00–20.0	
		3 days postpartum	372	TSH (mIU/L)	2.37	0.15–9.51	TT3 (nmol/L)	2.75	1.32–4.66	TT4 (nmol/L)	144	77.0–232	Positive (> 34 IU/L): 4%
							FT3 (pmol/L)	4.50	2.72–6.77	FT4 (pmol/L)	13.0	8.00–19.0	
		6 weeks postpartum	374	TSH (mIU/L)	1.39	0.06–6.54	TT3 (nmol/L)	1.70	1.15–2.53	TT4 (nmol/L)	97.0	63.0–153	Positive (> 34 IU/L): 4%
							FT3 (pmol/L)	4.63	3.14–6.48	FT4 (pmol/L)	14.0	10.0–25.0	
[44]	Wang Taiwan 2014	Week 28–40 (3rd trimester)	274–285	TSH (mIU/L)	1.76		TT3 (nmol/L)*	2.45		TT4 (nmol/L)*	143.7		
										FT4 (pmol/L)*	7.3		
[45]	Reardon Canada 2019	< 13 weeks	167	TSH (mIU/L)*^, e^	1.18	0.15–3.21^c^	FT3 (pmol/L)	4.62	3.72–5.58 ^c^	FT4 (pmol/L)	14.9	11.8–19.6 ^c^	High (> 9 IU/mL): 5%
		14–26 weeks	487	TSH (mIU/L)*^, e^	1.38	0.57–3.25 ^c^	FT3 (pmol/L)	4.53	3.69–5.51 ^c^	FT4 (pmol/L)	14.0	11.6–17.5 ^c^	High (> 9 IU/mL): 15%
		27–40 weeks	465	TSH (mIU/L)*^, e^	1.28	0.52–2.45 ^c^	FT3 (pmol/L)	4.36	3.62–5.41 ^c^	FT4 (pmol/L)	12.6	10.0–16.6 ^c^	High (> 9 IU/mL): 15%
		3 months postpartum	479	TSH (mIU/L)*^, e^	1.14	0.07–2.28 ^c^	FT3 (pmol/L)	4.13	3.30–5.30 ^c^	FT4 (pmol/L)	14.0	11.6–19.4 ^c^	High (> 9 IU/mL): 14%
[46]	Yang China 2016	1–2 days before delivery (3rd trimester)	123–157	TSH (mIU/L)	3.03	0.26–12.77	TT3 (nmol/L)	2.54	0.77–3.86	TT4 (nmol/L)	140.00	71.42–288.30	
							FT3 (pmol/L)	3.82	1.76–5.41	FT4 (pmol/L)	11.89	7.45–17.49	
[47]	Xiao Faroe Island 2019	Week 34 (3rd Trimester)	172	TSH (mIU/L)*^, f^	1.34^b^	0.23–3.61	FT3 (pmol/L)	4.25 ^b^	2.94–6.62	TT4 (nmol/L)	121.08 ^b^	61.00–224.00	
							T3RU	0.66 ^b^	0.51–1.01	FT4 (pmol/L)	8.18 ^b^	5.49–14.70	
										FT4Index (IU/L)	80.47 ^b^	53.00–164.00	

TT3 (total triiodothyronine), FT3 (free triiodothyronine), TT4 (total thyroxine), FT4 (free thyroxine), T3RU (Free triiodothyronine resin uptake), *: Converted values, for TSH all values are converted to mIU/L, for TT3 and TT4 all values are converted to nmol/L, for FT3 and FT4 all values are converted to pmol/L, ^a^: 95% confidence interval, ^b^: Geometric mean, ^c^: Range defined as the 5th percentile and 95th percentile of the sample population, ^d^: In the article the value reported were 0.99 ng/mL, converted to pmol/L giving 1274.3 pmol/L; other FT4 values from the same cohort (Minatoya et al. 2017) have reported similar values in ng/dL. We believe that the correct unit is 0.99 ng/dL (=12.7 pmol/L), which gives normal FT4 levels, ^e^: Values were reported as mIU/mL in tables, however other places in the article the more used μIU/mL or IU/L were used, we choose to interpret the values as μIU/mL as the values are in the normal range and comparable with values reported from other studies. ^f^: Values were reported as IU/L, we choose to interpret the values as μIU/mL / mIU/L as the values are then in the normal range and comparable with values reported from other studies. #: Aarhus University Hospital, Aarhus, Denmark

**Table 6:** Infant TH levels

				TSH			T3			T4		
Reference	Author Country Year	Time point of TH measurement	Number		Median	Range		Median	Range		Median	Range
Normal reference range		*1–7 days*✦		TSH (mIU/L) ✦	*Lower limit: 0.13–1.79* *Upper limit: 9.23–57.2*			*–*	*–*	FT4 (pmol/L) ✦	*Lower limit: 8.9–20.3* *Upper limit: 26.8–46.6*	
[38]	Kato Japan 2016	4–7 days postpartum (heelprick)	392	TSH (mIU/L)*	2.2	LOD–7.9 ^a^				FT4 (pmol/L) *^, d^	26.1	17.9–37.2 ^a^
[39]	Preston USA 2018	3 days postpartum (heel prick)	480							TT4 (nmol/L)*	222.7	47.6–459.5
[44]	Wang Taiwan 2014	At delivery (cord blood serum)	92–116	TSH (mIU/L)*	6.65		TT3 (nmol/L)*	0.77		TT4 (nmol/L)*	107.7	
										FT4 (pmol/L)*	9.40	
[46]	Yang China 2016	At delivery (cord blood serum)	146–157	TSH (mIU/L)*	9.44	1.26–57.01	TT3 (nmol/L)	0.90	0.56–3.16	TT4 (nmol/L)	129.70	69.52–232.20
							FT3 (pmol/L)	2.01	1.11–4.45	FT4 (pmol/L)	15.88	9.44–21.26
[47]	Xiao Faroe Island 2019	At delivery (cord blood serum)	153	TSH (mIU/L)*^, e^	6.98^b^	2.03–27.40	FT3 (pmol/L)	2.35^b^	2.00–5.54	T4 (nmol/L)	129.07^b^	68.00–205.00
							T3RU	0.83^b^	0.54–1.05	FT4 (pmol/L)	11.93^b^	0.38–16.60
										FT4I (IU/L)	107.60^b^	65.00–191.00
[48]	Dufour Belgium 2018	3 days postpartum (heel prick)	214	TSH (mIU/L)	4.85	Max: 16.6						
[49]	Shah-Kulkarni Korea 2016	At delivery (cord blood serum)	279	TSH (mIU/L)*	8.3	1.2–49.6	TT3 (nmol/L)*	0.88	0.59–2.22	TT4 (nmol/L)*	108.1	61.8–168.6
[50]	De Cock Netherland 2014	4–7 days postpartum (heel prick)	Boys: 52							TT4 (nmol/L)	85.6	42–138
			Girls: 31							TT4 (nmol/L)	89.6	68–117
[51]	Tsai Taiwan 2017	At delivery (cord blood plasma)	118	TSH (mIU/L)*	2.92 ^c^	0.008–14.1	TT3 (nmol/L)*	0.46^c^	76.3–202.8	TT4 (nmol/L)*	115.6 ^c^	164.8–1530.5
							FT3 (pmol/L)*	2.29 ^c^	1.54–5.7	FT4 (pmol/L)*	15.4 ^c^	10.3–29.6
[52]	Aimuzi China 2019	At delivery (cord blood serum)	568	TSH (mIU/L)	4.66	1.73–28.87	FT3 (pmol/L)	2.03	< LOD-4.29	FT4 (pmol/L)	13.48	< LOD-18.44

TT3 (total triiodothyronine), FT3 (free triiodothyronine), TT4 (total thyroxine), FT4 (free thyroxine), FT4I (free thyroxine index); T3RU (Free triiodothyronine resin uptake), ✦: Correct to Onsesveren et al 2017; 10.21820/23987073.2017.6.22 based on review of 35 studies of TSH and FT4 in childhood, *: Converted values, for TSH all values are converted to mIU/L, for TT3 and TT4 all values are converted to nmol/L, for FT3 and FT4 all values are converted to pmol/L, ^a^: 95% confidence interval,^b^: Geometric mean, ^c^: Mean, ^d^: In the article the value reported were 2.03 ng/mL, converted to pmol/L giving 2613 pmol/L; other FT4 values from the same cohort (Minatoya et al. 2017) have reported similar values in ng/dL. We believe that the correct unit is 2.03 ng/dL (=26.1 pmol/L), which gives more normal FT4 levels, ^e^: Values were reported as IU/L, we choose to interpret the values as μIU/mL / mIU/L as the values are then in the normal range and comparable with values reported from other studies

As shown in Table 6, infant TSH median levels were in general within the normal reference range (i.e. being higher than the lower limit but lower than the upper limit). Moreover, as expected for the T3 (TT3 and FT3), the TT3 levels were higher than FT3. Similar values of T4 (TT4 and FT4) levels were also seen among the studies and FT4 levels were mainly within the lower limit range (Table 6).

### Association between PFAAs exposure and thyroid hormone outcomes

The associations between PFAAs exposure and TH outcomes are outlined by the following order: i) maternal TSH (Table 7), ii) maternal T4 and T3 (Table 8), iii) infant TSH (Table 9) and iv) infant T4 and T3 (Table 10). Maternal TH estimates were assessed by the trimester in which they were measured. Infant TH estimates were assessed upon stratification by sex (Suppl. Tables 1 and 2).

#### Maternal PFAAs exposure and TSH levels (Table 7)

Two out of nine studies (Table 7) showed a significantly positive association between maternal PFHxS concentrations and TSH levels in the 3rd trimester [44] or at multiple time points [45]. Two out of ten studies found an inverse association between PFOS and maternal TSH levels (in the 1st [38] and 3rd trimester [46]). However, two other studies found PFOS significantly positively associated with maternal TSH levels at the 2nd trimester [41] and at multiple time-points [43], respectively. In fact, women with the highest PFOS concentration had a significant 24% higher TSH levels than those with the lowest PFOS concentration at multiple time points (data not shown) [43].

**Table 7:** Association of maternal PFAAs exposure and maternal TSH by trimester (adjusted results)

Reference	Author Country Year	Effect estimate	PFHxS	PFOS	PFOA	PFNA	PFDA	PFUnA	PFDoA
**TH measured during 1st Trimester**									
[38]	Kato Japan 2016	Linear regression: Adjusted β (p value)	–	↓: − 0.214 (< 0.001)*	↑: 0.039 (0.478)	–	–	–	–
[39]	Preston USA 2018	Difference in maternal TH levels per quartile (Q2–4 vs. Q1) of PFAAs concentrations (95% CI)	↑: Q2: 7.66 (− 13.1, 33.4)	↓: Q2: − 6.91 (− 25.1, 15.7)	↑: Q2: 16.0 (− 7.08, 44.8)	↑: Q2: 22.6 (− 2.79, 54.5)	–	–	–
			↓: Q3: − 8.58 (− 26.7, 14.0)	↓: Q3: − 5.73 (− 24.5, 17.6)	↑: Q3: 10.1 (− 12.0, 37.7)	↑: Q3: 12.1 (− 9.20, 38.3)	–	–	–
			↑: Q4: 3.27 (− 16.9, 28.4)	↓: Q4: − 5.04 (− 24.0, 18.6)	↑: Q4: 8.35 (− 14.1, 36.6)	↑: Q4: 6.46 (− 13.6, 31.1)	–	–	–
[40]	Inoue Denmark 2019	Relative percentage difference per IQR increase	↑: 1.02 (0.96, 1.08)	↑: 1.04 (0.96; 1.14)	↑: 1.01 (0.93; 1.1)	↑: 1.01 (0.95; 1.08)	↑: 0.99 (0.93; 1.05)	–	–
**TH measured during 2nd Trimester**									
[42]	Webster Canada 2014	IQR increase in maternal PFAAs concentrations: Adjusted β (95% CI)	↑: 0.01 (− 0.05, 0.07)	↑: 0.1 (− 0.03, 0.2)	↑: 0.1 (− 0.05, 0.3)	**↑: 0.2** (0.01, 0.3)*	–	–	–
[41]	Wang Norway 2013	Linear regression: Adjusted β (95% CI)	↑: 0.013 (− 0.043, 0.070)	**↑: 0.008** (0.001, 0.016)*	↓: − 0.0001 (− 0.045, 0.044)	↑: 0.165 (− 0.023, 0.353)	↑: 0.060 (− 0.458, 0.578)	↑: 0.080 (− 0.200, 0.360)	–
**TH measured during 3rd Trimester**									
[44]	Wang Taiwan 2014	Linear regression: Adjusted β (95% CI)	**↑: 0.105** (0.002, 0.207)*	↓: − 0.005 (− 0.024, 0.013)	↑: 0.011 (− 0.057, 0.078)	↑: 0.033 (− 0.046, 0.112)	↑: 0.004 (− 0.037, 0.045)	↑: 0.011 (− 0.009, 0.030)	↑: 0.365 (− 0.215, 0.944)
[46]	Yang China 2016	Spearmans partial correlation analysis. *p* < 0.05. Maternal exposure used.	↓: − 0.154	↓: − 0.261*	↓: − 0.124	↓: − 0.170*	↓: − 0.216*	↓: − 0.202*	↓: − 0.231*
[47]	Xiao Faroe Island 2019	Percent change in thyroid hormone levels per doubling of PFAA concentrations (95% CI)	↑: 7.4 (− 11.8, 30.9)	↑: 16.4 (−7.5, 46.5)	↑: 12.6 (−4.5, 32.8)	↑ 20.1 (− 5.3, 52.3)	↑: 13.9 (−9.0, 42.6)	↓: − 0.3 (− 15.4, 17.4)	↓: −2.5 (− 9.2, 4.7)
**TH measured during multiple timepoints**									
[43]	Berg Norway 2015	Mixed effects model estimated mean differences in thyroid hormone concentrations over time (2nd trimester, at birth and 6 weeks postpartum). Change across exposure quartiles (Q1 reference). NS: results not stated.		↑: Q2: 0.18 (0.06, 0.31)					
			NS	**↑: Q3: 0.26** (0.13, 0.40)*	NS	NS	NS	NS	–
				**↑: Q4: 0.35** (0.21, 0.50)*					
[45]	Reardon Canada 2019	Overall main effect of PFAAs on TH (all timepoints) from mixed effects models: Adjusted β (*p*-value)	↑: 0.144 (0.008)*	**↑:** 0.082 (0.069)	**↑:** 0.007 (0.368)	**↑:** 0.005 (0.810)	**↑:** 0.023 (0.647)	**↓:** −0.024 (0.901)	–

TSH (thyroid stimulating hormone), ↓ decreasing, ↑ increasing, bold format and star* indicate significant results *P* < 0.05, *NS*: no result stated, −: PFAAs not examined. Q1 (1st quartile), Q2 (2nd quartile), Q3 (3rd quartile), Q4 (4th quartile), IQR (interquartile range)

PFAAs include: *PFOS* Perfluorooctane sulfonate, *PFOA* Perfluorooctanoate, *PFHxS* Perfluorohexane sulfonate, *PFNA* Perfluorononanoic acid, *PFDA* Perfluorodecanoic acid, *PFUnA* Perfluoroundecanoic acid, *PFDoA* Perfluorododecanoic acid

No significant associations were found between maternal PFOA concentration and TSH levels (Table 7).

Among nine studies, one study found a positive association between PFNA concentration and maternal TSH levels (Table 7) in the 2nd trimester [42], while another study found an inverse association within 3rd trimester TSH [46].

One out of seven studies found a significant inverse association between PFDA and maternal 3rd trimester TSH levels (Table 7) [46].

The association between maternal PFUnA and TSH was examined in six studies (Table 7), and Yang et al. 2016 [46] observed a significantly inverse association in the 3rd trimester. In the same study [46] the maternal PFDoA concentration and TSH level were as well found inversely associated in the 3rd trimester (Table 7).

#### Maternal PFAAs exposure and T4 and T3 by trimester (Table 8)

Maternal T4 and T3 were assessed by nine studies (Table 8). Significantly inverse associations were observed between maternal FT4 effect estimates (FT4I) and PFHxS levels in the 1st trimester [39] and at multiple time points [45]. Although positive tendencies were found, none of the results were significant for PFOS exposure and maternal T4 and T3 levels (Table 8). Out of nine studies, investigating the association maternal PFOA concentration with T4 and T3 levels (Table 8), one study found a significantly inverse association with maternal 1st trimester FT4I levels when comparing the 2nd and 4th quartiles with the lowest exposure quartile [39]. Among the eight studies evaluating maternal PFNA concentrations and T4 and T3 levels (Table 8), one study found that PFNA concentrations inversely associated with maternal FT4 and TT4 levels at the 3rd trimester [44]. Among six studies (Table 8), one study reported a positive association of PFDA and maternal TT3 in the 3rd trimester [44], while another study observed a significantly negative association of high PFDA concentration (4th quartile) and TT3 at multiple time points [43]. Out of five studies assessing maternal T4 and T3 (Table 8), only one showed that FT3 measured at multiple time points decreased with high PFUnA concentrations (4th quartile) [43], while another study found inverse association between FT4 and TT4 and PFUnA exposure in the 3rd trimester [44]. Maternal T4 and T3 versus PFDoA exposure were assessed by three studies (Table 8); two studies observed a significantly inverse association with maternal 3rd trimester FT3, TT3, FT4 and TT4 levels [44, 46].

**Table 8:** Association of maternal PFAAs exposure and maternal T4 and T3 by trimester (adjusted results)

Reference	Author Country Year	Effect estimate	TH	PFHxS	PFOS	PFOA	PFNA	PFDA	PFUnA	PFDoA
**TH measured during 1st Trimester**										
[38]	Kato Japan 2016	Linear regression: Adjusted β (*p* value)	FT4	–	↑: 0.061 (0.236)	↑: 0.004 (0.946)	–	–	–	–
[39]	Preston USA 2018	Difference in maternal TH levels per quartile (Q2–4 vs. Q1) of PFAA concentrations (95% CI)	FT4Ic	↓ Q2: −0.25 (− 3.55, 3.16)	↓ Q2: − 0.56 (− 3.93, 2.93)	**↓ Q2: −4.68** (− 7.93,− 1.32)*	↓ Q2: − 1.94 (−5.48, 1.72)	**–**	–	–
				↓ Q3: − 0.35 (− 3.77, 3.18)	↓ Q3: − 1.41 (− 4.80, 2.09)	↓ Q3: −2.89 (− 6.23, 0.58)	↓ Q3: − 0.53 (− 3.79, 2.85)	**–**	–	–
				**↓ Q4: − 4.19** (− 7.43, − 0.85)*	↓ Q4: − 1.51 (− 4.92, 2.02)	**↓ Q4: − 6.52** (−9.86, − 3.06)*	↓ Q4: − 1.06 (− 4.27, 2.25)	**–**	–	–
			TT4	↓ Q2: − 0.23 (− 0.61, 0.14)	↑ Q2: 0.28 (− 0.10, 0.66)	↓ Q2: − 0.12 (− 0.51, 0.27)	↑ Q2: 0.02 (− 0.39, 0.43)	**–**	–	–
				↑ Q3: 0.10 (− 0.28, 0.49)	↑ Q3: 0.14 (− 0.25, 0.52)	↑ Q3: 0.01 (− 0.38, 0.40)	↑ Q3: 0.08 (− 0.29, 0.45)	–	–	–
				↓ Q4: − 0.34 (− 0.73, 0.04)	↑ Q4: 0.31 (− 0.08, 0.70)	↑ Q4: 0.02 (− 0.39, 0.43)	↓ Q4: −0.09 (− 0.45, 0.28)	–	–	–
[40]	Inoue Denmark 2019	Relative percentage difference per IQR increase	FT4	↑ 1.00 (0.98, 1.01)	↑ 1.00 (0.99, 1.02)	↑ 1.01 (0.99, 1.02)	↑ 1.00 (0.99; 1.01)	↑ 1.01 (1.00; 1.02)		
**TH measured during 2nd Trimester**										
[42]	Webster Canada 2014	IQR increase in maternal PFAA concentrations: Adjusted β (95% CI)	FT4	↓:-0.02 (− 0.1, 0.07)	↑: 0.03 (− 0.2, 0.2)	↓: − 0.06 (− 0.3, 0.2)	↓: − 0.03 (− 0.2, 0.2)	–	–	–
**TH measured during 3rd** **Trimester**										
[44]	Wang Taiwan 2014	Linear regression: Adjusted β (95% CI)	FT4	↓: − 0.010 (− 0.023, 0.003)	↑: 0.001 (− 0.002, 0.003)	↓: − 0.003 (− 0.012, 0.005)	**↓: − 0.019** (− 0.028, − 0.009)*	↓: − 0.001 (− 0.006, 0.005)	**↓: − 0.004** (− 0.007, − 0.002)*	**↓: − 0.132** (− 0.204, − 0.059)*
			TT4	↓:-0.130 (− 0.316, 0.057)	↑: 0.019 (− 0.016, 0.053)	↑: 0.011 (− 0.108, 0.130)	**↓: − 0.189** (− 0.333, − 0.046)*	↑: 0.047 (− 0.028, 0.123)	**↓: − 0.062** (− 0.097, − 0.026)*	**↓: − 1.742** (− 2.785, − 0.700)*
			TT3	↓:-0.002 (− 0.005, 0.001)	↑: 0.000 (− 0.002, 0.001)	↓:-0.000 (− 0.002, 0.009)	↓: − 0.001 (− 0.003, 0.002)	**↑: 0.002** (0.000, 0.003)*	↓: − 0.000 (− 0.001, 0.000)	↓: − 0.005 (− 0.022, 0.011)
[46]	Yang China 2016	Spearmans partial correlation analysis Maternal exposure used.	FT3	↑: 0.124	↑: 0.025	↑: 0.024	↓: − 0.063	↓: − 0.087	↓: − 0.121	↓: − 0.268*
			FT4	↑: 0.038	↓: − 0.057	↑: 0.000	↓: −0.072	↓: − 0.086	↓: − 0.062	↓: − 0.160*
			TT3	↑: 0.084	↑: 0.008	↑: 0.102	↓: −0.018	↓: −0.079	↓: − 0.097	↓: − 0.301*
			TT4	↑: 0.084	↑: 0.021	↑: 0.062	↓: −0.006	↓: −0.010	↑: 0.030	↓: − 0.160*
[47]	Xiao Faroe Island 2019	Percent change in thyroid hormone levels per doubling of PFAA concentrations (95% CI)	FT4I	↑: 4.4 (− 2.3, 11.6)	↓: −0.8 (− 8.2, 7.4)	↓: − 0.6 (− 6.1, 5.1)	↓: − 1.2 (− 8.9, 7.3)	↑: 0.2 (− 7.2, 8.1)	↑: 2.3 (− 3.1, 8.1)	↑: 0.2 (− 2.2, 2.6)
			FT3	↓: − 0.7 (−5.6, 4.5)	↑: 2.2 (− 3.6, 8.5)	↑: 3.1 (− 1.2, 7.6)	↓: − 2.7 (− 8.5, 3.6)	↓: − 1.4 (− 6.9, 4.5)	↓: − 1.8 (− 5.8, 2.4)	↑: 0.6 (− 1.3, 2.4)
			FT4	↑: 5.9 (− 0.2, 12.4)	↓: − 2.9 (− 9.4, 4.3)	↓: − 0.4 (− 5.4, 4.8)	↓: − 5.7 (− 12.4, 1.4)	↓: − 1.5 (− 8.1, 5.5)	↓: − 0.1 (− 5.0, 5.0)	↑: 0.2 (− 2.0, 2.4)
			TT4	↑: 0.4 (− 6.9, 8.2)	↑: 0.1 (−8.3, 9.3)	↑: 0.7 (− 5.5, 7.3)	↓: − 1.2 (− 9.9, 8.2)	↓: − 2.1 (− 10.1, 6.7)	↑: 0.4 (− 5.6, 6.9)	↑: 0.1 (− 2.6, 2.9)
			T3RU	↑: 4.0 (− 0.4, 8.5)	↓: − 0.9 (− 5.8, 4.3)	↓: − 1.3 (− 4.9, 2.3)	↑: 0.1 (− 5.1, 5.5)	↑: 2.2 (− 2.7, 7.5)	↑: 1.9 (− 1.7, 5.7)	↑: 0.1 (− 1.5, 1.7)
**TH measured during multiple timepoints**										
[43]	Berg Norway 2015	Mixed effects model estimated mean differences in thyroid hormone concentrations over time (2nd trimester, at birth and 6 weeks postpartum). Change across exposure quartiles (Q1 reference). NS: results not stated.	TT3	NS	NS	NS	NS	↓: Q2: − 0.04 (− 0.08. 0.04)	NS	
								↓: Q3: −0.05 (− 0.08, 0.00)		–
								**↓:Q4: −0.1** (− 0.14, − 0.06)*		
			FT3	NS	NS	NS	NS	NS	**↓ Q2: −0.08** (−0.15, − 0.00)*	
									**↓: Q3: − 0.09** (−0.16, − 0.01)*	–
									**↓ Q4: −0.18** (− 0.25, − 0.12)*	
			TT4	NS	NS	NS	NS	NS	NS	–
			FT4	NS	NS	NS	NS	NS	NS	–
[45]	Reardon Canada 2019	Overall main effect of PFAAs on TH (all timepoints) from mixed effects models: Adjusted β (*p*-value)	FT4	↓:-0.006 (0.034)*	**↓:**-0.000 (0.999)	**↓:**-0.002 (0.138)	**↓:**-0.004 (0.379)	**↓:**-0.010 (0.314)	**↓:**-0.037 (0.321)	–
			FT3	↑:0.000 (0.955)	**↑:-**0.003 (0.242)	**↑:**0.001 (0.535)	**↑:**0.004 (0.330)	**↑:**0.003 (0.736)	**↑:**0.014 (0.698)	–

TT3 (total triiodothyronine), FT3 (total triiodothyronine), TT4 (total thyroxine), FT4 (free thyroxine), FT4I (free thyroxine index), ↓ decreasing, ↑ increasing, bold format and star* indicate significant results *P* < 0.05, *NS*: results not stated, −: PFAAs not examined, Q1 (1st quartile), Q2 (2nd quartile), Q3 (3rd quartile), Q4 (4th quartile), IQR (interquartile range)

PFAAs include: *PFOS* Perfluorooctane sulfonate, *PFOA* Perfluorooctanoate, *PFHxS* Perfluorohexane sulfonate, *PFNA* Perfluorononanoic acid, *PFDA* Perfluorodecanoic acid, *PFUnA* Perfluoroundecanoic acid, *PFDoA* Perfluorododecanoic acid

#### PFAAs exposure and infant TSH level (Table 9)

PFHxS exposure elicited no significant association and no clear trend after examining four studies with infant TSH data (Table 9).

**Table 9:** Association of PFAAs exposure and infant TSH (adjusted results)

Reference	Author Country Year	Effect estimate	PFHxS	PFOS	PFOA	PFNA	PFDA	PFUnA	PFDoA
**Maternal PFAAs concentrations obtained during 3rd trimester**									
[38]	Kato Japan 2016	Linear regression: Adjusted β (*p* value)	–	↑: 0.177 (0.001) *	↓: − 0.014 (0.801)	–	–	–	–
[44]	Wang Taiwan 2014	Linear regression: Adjusted β (95% CI)	↑: 0.493 (− 1.449, 2.434)	↓: − 0.083 (− 0.292, 0.127)	↓: − 0.498 (− 1.464, 0.468)	↓: − 0.361 (− 0.955, 0.234)	↓: −3.505 (−7.821, 0.812)	↓: − 0.083 (− 0.232, 0.066)	↓: − 1.539 (−6.582, 3.503)
[46]	Yang China 2016	Spearmans partial correlation analysis. *p* < 0.05.	↓: −0.034	↓: − 0.119	↓: − 0.057	↓: − 0.105	↓: − 0.145	↓: − 0.094	↑: 0.016
[47]	Xiao Faroe Island 2019	Percent change in thyroid hormone levels per doubling of PFAA concentrations (95% CI)	↑: 11.5 (− 10.4, 39.0)	↑: 39.7 (7.9, 80.9)*	↑: 23.1 (1.9, 48.6)*	↑: 53.3 (18.2, 99.0)*	↑: 31.3 (2.2, 68.4)*	↑: 7.6 (− 10.2, 28.8)	↑: 0.2 (− 7.7, 8.7)
**Cord blood serum/plasma PFAAs concentrations**									
[48]	Dufour Belgium 2018	Multivariate regression: Adjusted β (*p* value)	–	↓: −0.028 (0.679)	↓: −0.094 (0.196)	↓: − 0.129 (0.064)	–	–	–
[49]	Shah-Kulkarni Korea 2016	Linear regression: Adjusted β (95% CI)	↓: − 1.09 (−2.60, 0.41)	↓: −0.15 (− 1.03, 0.73)	↓: − 0.79 (− 2.13, 0.55)	↓: − 0.83 (− 1.95, 0.28)	↓: − 1.01 (− 2.65, 0.62)	↓: −0.62 (− 2.01, 0.76)	↓: −1.29 (− 3.28, 0.69)
[51]	Tsai Taiwan 2017	Linear regression: Adjusted β (95% CI)	–	**↑: 0.346** (0.101, 0.591)*	↑: 0.059 (− 0.136, 0.254)	↑: 0.045 (− 0.051, 0.142)	–	↑: 0.077 ( − 0.063, 0.216)	–
[52]	Aimuzi China 2019	Sparse partial least squares (SPLS): Adjusted β (95% CI)	#	↓: **− 0.012** (− 0.019,-0.005)*	#	↓: **− 0.012** (− 0.019, − 0.005)*	↓: **−0.012** (− 0.02,-0.005)*	↓: **− 0.013** (− 0.021,-0.006)*	↓: **−0.013** (− 0.023,-0.006)*

TSH (thyroid stimulating hormone), ↓ decreasing, ↑ increasing, bold format and star* indicate significant results *P* < 0.05, −: PFAAs not examined, # indicates association was not selected in SPLS model

PFAAs include: *PFOS* Perfluorooctane sulfonate, *PFOA* Perfluorooctanoate, *PFHxS* Perfluorohexane sulfonate, *PFNA* Perfluorononanoic acid, *PFDA* Perfluorodecanoic acid, *PFUnA* Perfluoroundecanoic acid, *PFDoA* Perfluorododecanoic acid

Evaluating the PFOS exposure data in the 3rd trimester or cord blood, the infant TSH levels were examined in eight studies (Table 9). Maternal 3rd trimester [38, 47] and cord blood PFOS concentrations [51] were significantly positively associated with infant TSH levels (Table 9). But, an inverse association was found in another study assessing PFOS in cord blood and infant TSH [52]. Upon stratification by infant sex, three studies found positive associations among boys [38]: β_male_ = 0.205, *p* = 0.014, [47]: % change per doubling in PFOS = 39.5, 95%CI (0.4, 94.1) [51]: β_male_ = 0.333, 95%CI (0.012, 0.678)] (Suppl. Table 1a). While among female infants, one study found positive [38] [β_female_ = 0.173, *p* = 0.021] and one inverse [52] [β_female_ = − 0.016, 95%CI (− 0.032, − 0.002)] association, respectively (Suppl. Table 1b).

Using cord blood or maternal 3rd trimester PFOA exposure, the association of PFOA with infant TSH was examined in eight studies (Table 9). A positively significant association was found in one study with maternal 3rd trimester PFOA concentrations [47], while negative non-significant tendencies were seen in several other studies [38, 44, 46, 48, 49].

Infant TSH was assessed upon PFNA exposure in seven studies (Table 9), and a positive association was seen with maternal PFNA concentrations at the 3rd trimester [47], while an inverse association was found with cord blood PFNA concentrations [52]. When stratifying by infant sex (Suppl. Table 1a and b), for males, a significantly positive association with maternal 3rd trimester PFNA concentrations [% change per doubling in PFNA_male_ = 60.6, 95%CI (15.0, 124.6)] [47] and an inverse association with cord blood PFNA concentrations [β_male_ = − 0.166, *p* = 0.018] [48] was found. Among girls, a similar pattern for TSH was seen with a positive association with maternal 3rd trimester PFNA concentrations [% change per doubling in PFNA_female_ = 46.7, 95%CI (5.9, 103.2)] [47] and an inverse association with cord blood PFNA concentrations [β_female_ = − 1.69, 95%CI (− 3.31, − 0.08)] [49].

Infant TSH (Table 9) was found positively associated with maternal 3rd trimester PFDA concentrations [47], while an inverse association was found with cord blood PFDA concentrations [52]. Other studies also showed decreasing tendencies (in maternal and cord blood), however the associations were non-significant [44, 46, 49]. When stratifying by infant sex (Suppl. Table 1a and b), infant TSH was positive associated with maternal PFDA in 3rd trimester concentrations among boys [% change per doubling in PFDA_male_ = 37.0, 95%CI (0.5, 86.8)] [47].

The relation between infant TSH level and PFUnA concentration were examined in six studies (Table 9) and one study showed a significantly inverse association with cord blood PFUnA concentrations [52].

Among five studies that examined cord blood PFDoA concentration and infant TSH level (Table 9), one study found a significantly inverse association [52]. After stratification by infant sex, the inverse association was only significant among boys [52] [β_male_ = − 0.062, 95%CI (− 0.108; − 0.017)] (Suppl. Table 1a).

#### PFAAs exposure and infant T4 and T3 levels (Table 10)

PFHxS exposure and infant T4 and T3 levels were evaluated by six studies (Table 10) and a study found high maternal PFHxS levels (4th quartile) were significantly inversely associated with infant TT4 levels [39]. After stratification by infant sex, this TT4 association remained significant only among males [Q4: β_male_ = − 2.51, 95%CI (− 3.99, − 1.04)] [39] (Suppl. Table 2a). Whereas, cord blood PFHxS levels were associated with increased female infant TT3 levels [β_female_ = 4.28, 95%CI (0.39, 8.17)] [49] (Suppl. Table 2b).

**Table 10:** Association of PFAAs exposure and infant T4 and T3 (adjusted results)

Reference	Author Country Year	Effect estimate	TH	PFHxS	PFOS	PFOA	PFNA	PFDA	PFUnA	PFDoA
**Maternal PFAAs concentrations obtained during 1st Trimester**										
[39]	Preston USA 2018	Difference in infant TH levels per quartile (Q2–4 vs. Q1) maternal PFAAs concentrations (95% CI)	TT4	↓ Q2: −0.76 (− 1.78, 0.26)	↓ Q2: − 0.63 (− 1.64, 0.37)	↓ Q2: − 0.44 (− 1.46, 0.59)	↓ Q2: − 0.34 (− 1.42, 0.73)	–	–	–
				↓ Q3: − 0.96 (− 1.94, 0.03)	↓ Q3: − 0.36 (− 1.39, 0.67)	↓ Q3: − 0.35 (− 1.39, 0.69)	↓ Q3: −0.60 (− 1.52, 0.31)	–	–	–
				↓ Q4: −1.06 (− 2.06, − 0.06)*	↓ Q4: − 1.10 (− 2.13, − 0.07)*	↓ Q4: −1.13 (− 2.21, − 0.06)*	↓ Q4: − 0.53 (− 1.56, 0.49)	–	–	–
**Maternal PFAAs concentrations obtained during 3rd Trimester**										
[38]	Kato Japan 2016	Linear regression: Adjusted β (*p* value)	FT4	–	↓: − 0.043 (0.452)	↑: 0.003 (0.960)	–	–	–	–
[44]	Wang Taiwan 2014	Linear regression: Adjusted β (95% CI)	FT4	↓: − 0.030 (− 0.098, 0.039)	↑: 0.001 (− 0.006, 0.008)	↓: − 0.029 (− 0.062, 0.004)	↑: 0.001 (− 0.021, 0.023)	↑: 0.020 (− 0.124, 0.164)	↑: 0.002 (− 0.004, 0.007)	↓: −0.009 (− 0.183, 0.165)
			TT4	↑: 0.002 (−0.495, 0.500)	↑: 0.032 (−0.024, 0.087)	↑: 0.128 (− 0.094, 0.350)	↓: − 0.213 (− 0.384, − 0.042)*	↓: −0.513 (− 1.732, 0.706)	↓: − 0.052 (− 0.095, − 0.010)*	↓:-1.920 (− 3.345, − 0.495)*
			TT3	↓: − 0.001 (− 0.007, 0.004)	↑: 0.000 (− 0.000, 0.001)	↓: −0.001 (− 0.004, 0.001)	↓: − 0.002 (− 0.004, − 0.001)*	↓: −0.017 (− 0.028, − 0.005)*	↓: −0.001 (− 0.001, − 0.0002)*	↓: −0.022 (− 0.035, − 0.009)*
[46]	Yang China 2016	Infant TH vs maternal PFAAs Spearmans partial correlation analysis. *p* < 0.05.	FT3	↑: 0.125	↑: 0.191*	↓: −0.105	↑: 0.094	↑: 0.154	↑: 0.108	↑: 0.116
			FT4	↓: −0.016	↑: 0.053	↓: −0.045	↓: − 0.023	↑: 0.027	↑: 0.033	↑: 0.011
			TT3	↑: 0.108	↑: 0.170*	↓: −0.055	↑: 0.118	↑: 0.188*	↑: 0.113	↑: 0.071
			TT4	↑: 0.111	↑: 0.172*	↑: 0.107	↑: 0.147	↑: 0.181*	↑: 0.172*	↑: 0.058
[47]	Xiao Faroe Island 2019	Percent change in thyroid hormone levels per doubling of PFAA concentrations (95% CI)	FT4I	↑: 0.3 (−6.2, 7.3)	↑: 6.7 (−1.5, 15.6)	↑: 2.6 (− 3.1, 8.9)	↑: 8.2 (−0.3, 17.4)	↑: 8.4 (0.5, 17.0)*	↑: 3.1 (−2.4, 9.0)	↓: −0.6 (− 3.1, 1.9)
			FT3	↓: −2.4 (−9.2, 4.8)	↓: −0.8 (− 9.1, 8.1)	↑: 0.5 (− 5.6, 6.9)	↑: 0.9 (− 7.6, 10.3)	↑: 1.1 (− 6.9, 9.7)	↑: 2.2 (− 3.6, 8.3)	↓: − 1.5 (− 4.0, 1.2)
			FT4	↑: 14.2 (− 2.6, 33.8)	↑: 15.6 (−4.5, 40.1)	↑: 1.9 (− 11.5, 17.2)	↑: 6.0 (− 13.1, 29.3)	↑: 7.6 (− 10.7, 29.4)	↓: − 0.4 (− 12.7, 8.3)	↑: 1.6 (− 4.2, 1.2)
			TT4	↓: − 2.5 (− 9.1, 4.6)	↑: 2.5 (− 5.7, 11.6)	↑: 1.9 (− 4.1, 8.3)	↑: 4.1 (− 4.5, 13.5)	↑: 5.4 (− 2.8, 14.3)	↑: 0.6 (− 5.1, 6.5)	↓: −1.6 (− 4.1, 1.0)
			T3RU	↑: 2.8 (− 0.6, 6.3)	↑: 3.6 (− 0.5, 7.8)	↑: 0.6 (− 2.4, 3.6)	↑: 3.7 (− 0.6, 8.0)	↑: 2.7 (− 1.1, 6.8)	↑: 2.4 (− 0.4, 5.2)	↑: 0.8 (− 0.4, 2.0)
**Cord blood serum/plasma PFAAs concentrations**										
[49]	Shah-Kulkarni Korea 2016	Linear regression: Adjusted β (95% CI)	TT3	↑: 1.81 (− 0.66, 4.28)	↑: 0.65 (− 0.80, 2.10)	↓: − 0.01 (− 2.22, 2.20)	↑: 0.07 (− 1.74, 1.92)	↑: 2.40 (− 0.27, 5.09)	↑: 1.06 (− 1.21, 3.34)	↑: 1.69 (− 1.57, 4.96)
			TT4	↑: 0.03 (− 0.26, 0.32)	↑: 0.14 (− 0.03, 0.31)	↑: 0.001 (− 0.26, 0.26)	↑: 0.04 (− 0.17, 0.26)	↑: 0.13 (− 0.18, 0.45)	↓: − 0.02 (− 0.29, 0.24)	↓: − 0.07 (− 0.46, 0.31)
[50]	De Cock Netherland 2014	Linear regression coefficients (95% CI): Change across quartiles (Q1 reference) Infant sex analysed separately	Male TT4		↓: Q2: −7.9 (− 31.56, 15.74)	↑: Q2: 7.9 (− 18.04, 33.92)				
				–	↓: Q3: − 16.5 (− 40.32, 7.34)	↓: Q3: − 2.1 (− 20.94, 16.78)	–	–	–	–
					↓: Q4: −9.6 (−32.57, 13.31)	↑: Q4: 6.2 (− 16.08, 28.50)				
			Female TT4		↓: Q2: −1.3 (−30.45, 27.94)	↓: Q2: −5.9 (− 26.75, 14.94)				
				–	↑: Q3: 4.5 (− 25.95, 34.92)	↑: Q3: 11.8 (− 19.08, 42.72)	–	–	–	–
					↑: Q4: 15.9 (− 10.67, 42.40)	**↑: Q4: 38.6** (13.34, 63.83)*				
[51]	Tsai Taiwan 2017	Linear regression: Adjusted β (95% CI)	TT4	–	**↓: −0.458** (− 0.916,-0.001)*	↓:-0.031 (− 0.404, 0.342)	↑: 0.067 (− 0.252, 0.119)	–	↑: 0.045 (− 0.223,0.313)	–
			TT3	–	↑: 0.027 (− 0.072, 0.125)	↑: 0.025 (− 0.054, 0.103)	↓: − 0.03 (− 0.069, 0.009)	–	↑: 0.048 (− 0.008, 0.104)	–
[52]	Aimuzi China 2019	Sparse partial least squares (SPLS): Adjusted β (95% CI)	FT4	#	#	#	#	#	#	**↑: 0.19** (0.063, 0.304)*
			FT3	#	**↑: 0.059** (0.023, 0.100)*	#	↑: 0.024 (−0.027, 0.071)	↓: − 0.008 (− 0.04, 0.026)	↑: 0.018 (− 0.034, 0.077)	**↓: − 0.153** (− 0.212, − 0.106)*

TT3 (total triiodothyronine), FT3 (total triiodothyronine), TT4 (total thyroxine), FT4 (free thyroxine), FT4I (free thyroxine index), ↓ decreasing, ↑ increasing, bold format and star* indicate significant results *P* < 0.05, −: PFAAs not examined, # indicates association was not selected in SPLS model, Q1 (1st quartile), Q2 (2nd quartile), Q3 (3rd quartile), Q4 (4th quartile), IQR (interquartile range)

*PFOS* Perfluorooctane sulfonate, *PFOA* Perfluorooctanoate, *PFHxS* Perfluorohexane sulfonate, *PFNA* Perfluorononanoic acid, *PFDA* Perfluorodecanoic acid, *PFUnA* Perfluoroundecanoic acid, *PFDoA* Perfluorododecanoic acid

The assessement of PFOS exposure and infant T4 and T3 found in four studies significant associations (Table 10). High PFOS concentration in the 1st trimester (4th quartile) [39] and in cord blood [51] was inversely associated with infant TT4 levels (Table 10). In contrast, maternal 3rd trimester PFOS concentration [46] was significantly and positively associated with increased infant TT3, FT3 and TT4 levels. Likewise, infant cord blood PFOS concentrations [52] was positively associated with FT3 levels (Table 10). Upon stratification by infant sex, the significant inverse associations with TT4 remained only among boys ([Q4 β_male_ = − 2.20, 95%CI (− 3.74; − 0.66)] and [β_male_ = − 0.667, 95%CI (− 1.28; − 0.05)]) [39, 51] (Suppl. Table 2a), while one study found a positive association with FT4I among girls [% change per doubling in PFOS_female_ = 13.2, 95%CI (0.9, 27.1)] [47] (Suppl. Table 2b).

One study, out of nine, found infant TT4 levels (Table 10) to be significantly and inversely associated with higher maternal PFOA concentration (4th quartile) in the 1st trimester [39]. After stratification by sex, the significance for TT4 remained only among boys [Q4 β_male_ = − 1.72, 95%CI (− 3.36; − 0.08)] [39] (Suppl. Table 2a). Another study found that cord blood PFOA concentration was positively associated with FT4 in males [52] [β_male_ = 0.062, 95%CI (0.024; 0.138)] (Suppl. Table 2a). Whereas, increased TT4 levels were found in girls with the highest cord blood PFOA concentration (4th quartile) [Q4 β_female_ = 38.6, 95%CI (13.34; 63.83)] [50] (Table 10, Suppl. Table 2b).

Infant T4 and T3 versus PFNA exposure was investigated in seven studies (Table 10) and one study found that infant TT3 and TT4 were negatively associated with maternal PFNA concentrations at 3rd trimester [44]. After stratification by sex, FT4 was found positively associated with cord blood PFNA concentration in boys [52] [β_male_ = 0.04, 95%CI (0.006, 0.081)] (Suppl. Table 2a).

Five studies investigated the infant T4 and T3 levels versus PFDA exposure (Table 10). Wang et al. 2014 demonstrated that maternal 3rd trimester PFDA concentration was inversely [44] and Yang et al. 2016 found a positively [46] association with infant TT3 levels. Likewise, infant TT4 and FT4I were positively associated with maternal 3rd trimester PFDA concentrations [46, 47]. After stratification by infant sex, FT4 was found positively associated with cord blood PFDA concentrations for males [52] [β_male_ = 0.043, 95%CI (0.016; 0.078)]. Moreover, a positive association between PFDA and FT4I in males was observed [% change per doubling in PFDA_male_ = 11.5, 95%CI (1.5; 22.6)] [47] (Suppl. Table 2a).

In two out of six studies significant data was found for exposure to PFUnA and association to infant T4 and T3 levels (Table 10). Maternal 3rd trimester PFUnA concentration was associated inversely with TT3 and TT4 infant levels in one study [44], and another study found increasing infant TT4 levels [46]. Tsai et al. found no effect in the total study population but a significant positive association when examining the 60th–89th percentile of cord blood PFUnA concentration with infant T3 levels [β = 0.197, 95%CI (0.031;0.364), *P* < 0.05] (data not shown) [51].

For PFDoA exposure versus infant T4 and T3 levels two of five studies found significant associations (Table 10). An inverse association with maternal 3rd trimester PFDoA was found in one study [44]. Cord blood PFDoA concentration was found negatively associated with infant FT3 but positively with FT4 [52]. After stratification by infant sex, FT4 was positively associated with cord blood PFDoA concentration in both boys and girls ([β_male_(FT4) = 0.54, 95%CI (0.019; 0.119)], [β_female_(FT4) = 0.174, 95%CI (0.019; 0.331)] [52], but for FT3, the inverse significant association remained only among girls [β_female_(FT3) = − 0.124, 95%CI (− 0.185; − 0.056)] [52] (Suppl. Table 2a and b).

## Discussion

This literature review suggests that PFAAs exposure can influence maternal TSH levels. Moreover, several PFAAs were inversely associated with maternal T4 and T3 levels. The studies reported some associations between PFAAs exposure and infant TH levels although the data was less conclusive.

### TSH

PFHxS, PFOS, PFNA, PFDA, PFUnA and PFDoA exposure seem to affect maternal TSH levels; however, only PFHxS, PFOS and PFNA were found significantly associated with TSH in more than one of the reviewed studies. Thus, five studies found significant increased maternal TSH levels upon exposure to PFOS, PFNA and PFHxS [41–45], which might indicate a (subclinical) hypothyroid maternal state. Two studies found significantly decreased maternal TSH levels with increasing PFOS, PFNA, PFDA, PFUnA and PFDoA concentrations [38, 46], which might indicate a hyperthyroid maternal state. None of the ten studies investigating the association between PFOA concentration and maternal TSH found any significant results [38–47]. However, it should be noted that all these PFAAs are often highly intercorrelated.

Three studies found increased TSH levels in infant upon PFOS exposure [38, 47, 51], while one study also found increased infant TSH levels with PFOA, PFNA and PFDA concentrations [47]. A single study found decreasing infant TSH with PFOS, PFNA, PFDA, PFUnA and PFDoA concentrations [52]. None of the studies investigating the association between PFHxS (*n* = 5) and infant TSH found any significant associations [44, 46, 47, 49, 52] and seven of the eight studies investigating the association with PFOA found non-significant results [38, 44, 46, 48, 49, 51, 52].

### T4 and T3

Five studies found decreasing maternal TT3, TT4, FT3, FT4 and FT4I levels associated with PFHxS, PFOA, PFNA, PFDA, PFUnA and PFDoA concentrations [39, 43–46]. Low levels of T4 and T3 (and high TSH) could indicate a hypothyroid maternal state. One study showed a positive association of maternal TT3 upon PFDA exposure [44].

Four studies found decreasing infant TT4, TT3 and FT3 with exposure to any of the investigated PFAAs [39, 44, 51, 52], and four studies found increasing infant FT3, FT4, FT4I, TT3 and TT4 levels with exposure to PFOS, PFOA, PFDA, PFUnA and PFDoA [46, 47, 50, 52].

In overall, most studies showed a positive association of maternal TSH and a possible negative association of maternal T4 and T3 upon PFAAs exposure. This indicates that pregnant women may be at risk of developing a hypothyroid state with increasing PFAAs exposure. Infant TH might also be affected, mainly increasing infant TSH levels with PFOS. More studies are needed to determine the direction of the association with infant T4 and T3 levels.

### Evaluation of TH across pregnancy trimesters with PFOS and PFOA exposure

During the 1st trimester, most studies on maternal TH [38, 39] (Table 7) showed that TSH was inversely related with PFOS, although, only one reached statistical significance [38]. Non-significant positive relationships were found with PFOA concentrations. PFOS and PFOA concentrations was mainly non-significantly but positively associated to maternal FT4 levels [38, 40] and TT4 levels [39] (Table 8). FT4I was inversely associated to PFOA but not significantly to PFOS [39].

In the 2nd trimester, studies on maternal TH [41, 42] (Table 7) showed that TSH was positively associated with PFOS exposure, being statistically significant in one study [41]. No consistency was found for the relation between PFOA and TSH, with both positive and inverse non-significant associations [41, 42]. Moreover, results on maternal T4 (Table 8) did not show any significant associations [42].

In the 3rd trimester, studies on maternal TH [44, 46, 47] (Table 7) demonstrated that TSH was significantly and inversely associated with maternal PFOS concentration in one study [46]. No consistent direction was found for PFOA exposure. For the maternal T4 and T3 levels [44, 46, 47] (Table 8), FT4, TT4, FT3 and TT3 were mainly positively, but non-significantly, associated with PFOS and PFOA.

Two studies conducted a longitudinal assessment of TH measurements across the trimesters and postpartum (Tables 2, 7 and 8) [43, 45]. Repeated measurements of maternal TSH were positively associated with PFOS exposure being significant in one study [43] (Table 7). A non-significantly inverse association was found for FT4, but positive for FT3 upon exposure to PFOS and PFOA [45](Table 8).

The trimester data evaluation showed negative associations between PFOS and maternal TSH in the 1st and 3rd trimester, but positive during the 2nd trimester (Table 7), whereas PFOA exposure did not elicite significant association to TSH in any of the trimesters. The trimester data evaluations for maternal T4 and T3 versus PFOS and PFOA concentrations showed inverse association between PFOA and FT4I in first trimester [39] (Table 8). Longitudinal TH studies found a positive association between TSH and PFOS [43] while data on PFOA exposure did not imply a strong association with maternal thyroid hormones. It must be noted that only few studies with information on each trimester were found, thus, the direction of the estimates might change when more studies are performed. Interestingly, it has been shown that associations of maternal TSH with exposure to PFHxS and branched PFOS isomers were strongest in early pregnancy (1st trimester) and weakened over subsequent trimesters (data not shown) [45].

### Stratifying by infant sex

Seven studies stratified on infant sex [38, 39, 47–49, 51, 52] (Suppl. Tables 1 and 2).

PFOS concentration was positively and significantly associated with TSH levels in boys [38, 47, 51] and girls [38]. PFNA was found to be inversely significantly associated with TSH levels in boys [48] and girls [49], while another study found positive associations in both genders [47] (Suppl. Table 1a-b). One study observed a negative association of TSH with cord blood PFOS exposure in girls and PFDoA in boys (Suppl. Table 1a-b) but they did not perform the statistical analysis on all PFAAs congeners in each sex due to their choice of statistical model (sparse partial least squares) [52].

In boys, TT4 levels were inversely associated with three PFAAs congeners (PFHxS, PFOS and PFOA) measured in maternal blood and cord blood samples (Suppl. Table 2a) [39, 51] and PNFA maternal concentrations were positively associated with FT4I [47]. In girls, TT3 levels were positively associated with cord blood PFHxS [49], and FT4I and TT4 levels positively associated with maternal PFOS and PFOA and cord blood PFOA (highest quartile) [47, 50] (Suppl. Table 2b). In boys, a study found significant positive associations for FT4 with several cord blood PFAAs (PFOA, PFNA, PFDA, PFDoA) concentration (Suppl.Table 2a), the analysis was however not performed in girls due to their choice of statistical model (sparse partial least squares) [52]. In the pooled infant analyses (Table 10) this study observed similar significant positive association between FT4 level, but inversely association with FT3 level, and cord blood PFDoA concentration [52].

The reviewed studies do not allow us to establish a distinct tendency or greater sensitivity regarding infant sex. Further studies must be conducted to state any conclusion on gender specific sensitivity for PFAAs exposure and TH disruption.

### Previous review literature

The possible link between PFAA exposure and thyroid outcomes in pregnant women was previously reviewed by Ballesteros et al. in 2017 [53]. They included 10 epidemiological studies on PFOS, PFOA, PFHxS and PFNA exposure during prenatal life, childhood and adolescence (up to 19 years), with sample size ranging from 40 to > 10.000 participants. In accordance to this current review, Ballesteros et al. suggested a positive association between PFHxS and PFOS and maternal TSH levels, as well as, between PFNA and TSH levels measured in boys aged > 11 years. Compared to Ballesteros et al. [53], the current review includes recent epidemiological studies (enrolment year: 1996–2016 vs. 1987–2013), more studies (*n* = 15 vs. *n* = 10), bigger sample size (participants > 100 vs. > = 43) and covers more PFAAs congeners (7 vs. 4), thus it might add a stronger study power and support to the previous observations. We also found stronger evidence of a positive relationship between PFAAs exposure (especially PFOS) with maternal TSH, and a possible inverse relationship with maternal T4 and T3 levels.

### Biological plausibility

PFAAs are endocrine disrupting compounds that impact on endogenous hormone homeostasis [54]. Different pathways have been suggested to explain thyroid disrupting effects: PFAAs may induce increased excretion of T4 by alteration of TBG due to competitive binding [55], and/or increased conversion of T4 to T3 by type 1 de-ionidase in hepatic cells, as well as increased hepatic metabolism of T4 [56]. PFAAs could furthermore alter the responsiveness of the HPT axis [57], as well as, alter the HPG homeostasis and their signaling pathways which might affect foetal growth [58, 59].

Several animal studies have shown reduced foetal growth upon PFAAs exposure [60–62]. Furthermore, animal studies have reported PFAAs disrupted HPT [60, 63, 64] and HPG-axis [65, 66]. However, comparison among studies is challenged because interspecies diversity (e.g. half-lives, excretion and plasmatic carrier proteins), PFAA congeners and levels of exposure, and extrapolation from animal studies to human risk assessment [64, 67].

Studies have suggested that thyroid and oestrogenic/androgenic pathways may be a part conjoined mechanisms. In vitro studies have shown that the HPT axis is affected by PFAAs exposure by inhibiting thyroid receptors, antagonizing thyroid cell proliferation [57]. Furthermore, PFOS and PFOA decreased TPO activity, blocking the ionizing process of thyroglobin [37], leading to reduced T4 and T3 levels. Cell proliferation of the TH-dependent rat pituitary GH3 cells was shown to be dependent on the involvement of the oestrogen receptor [68]. Moreover*,* in vitro studies have also shown alterations in the biosynthesis of oestrogens and interference with the oestrogen receptor, after exposure of PFNA, PFDA, PFHxS, PFOS and PFOA, due to their oestrogenic and anti-androgenic potential [2, 69–71]. Extracted PFAA mixtures from human serum samples also activated oestrogenic receptors [72, 73] and in an epidemiological study serum PFAAs mixture induced estrogenic activity in pregnant women was associated with significantly lower birth weight and birth length [58]. Most studies examined the effect of single PFAA congeners. More studies are needed for the combined cocktail effect of PFAAs mixtures and/or other POPs, which may have additively and/or synergistic actions [58, 73].

### PFAAs exposure and TH outcome assessment

All the reviewed studies determined PFAA concentrations, using small variations of the same methodology (liquid chromatography-tandem mass spectrometry), in mother and/or infant. Maternal biological matrix was either blood plasma or serum and for infant concentrations either cord blood plasma or serum. The LOD and LOQ values were different among studies. The percentages of samples below LOD/LOQ varied among studies, especially for PFHxS, PFNA, PFDA in maternal serum/plasma samples (Table 3) and for most PFAA congeners in cord blood (Table 4). Nevertheless, most selected studies handled the PFAA concentrations below LOD/LOQ with replacement by LOD/LOQ divided by the square root of 2. The sensitivity of the used methods might influence the estimated effect but the exposure levels is expected to be the main factor for the exposure - outcome associations.

Umbilical cord blood samples were also used to determine both infant exposure and thyroid function having the benefit being of representing the fetal environment. However, there are also some limitations of using cord blood for infant thyroid function assessment. Firstly, cord blood thyroid hormone levels likely reflects a combination of maternal and fetal hyroid hormones. Secondly, maternal and umbilical cord blood PFAAs concentrations are highly correlated [74, 75], and the ability of PFAAs to cross the placenta differs between compounds (i.e. PFOA passes more easily than PFOS [20, 75, 76].

Maternal PFAA concentrations might not be constant throughout pregnancy due to physiological hemodynamic changes. PFAAs concentrations decrease during pregnancy due to increased plasma volumes [77] and higher renal PFAAs excretion [78]. Thus, measurement of PFAAs in late pregnancy might not be directly compared with PFAAs concentration in 1st trimester [77]. Moreover, women are able to transfer some of their PFAAs burden to their offspring by placental [21, 79] and post-partum breast feeding [80]. This complicates comparison between studies with different pregnancy time points of exposure assessment [19]. Even more, one study measured the thyroid hormone outcome before exposure (PFAAs) [38], which could cause concern of causality, although some retrospective consistency for PFAAs exposure can be assumed due to their persistent nature (long half-lives in human serum) [15, 18]. However, a study showed the PFAA concentrations measured in the 1st and 3rd trimester were highly correlated (r > 0.64) [81] and argued that a single exposure measurement might be considered as a representative of serum levels during pregnancy.

In most of the studies, the exposure was evaluated as single congeners and not as the real mixture/combined exposure scenario. PFAAs are often highly inter-correlated. It is very diffecult to control for confounding by other PFAAs when assessing the effect of single PFAA congeners and interpret the association in light of the high mutual correlation among PFAA congeners. However, given the structural analogy and similar expected modes of action, PFAAs should be regulated as a chemical family rather than isolated compounds [82, 83]. In this regard, the complementation of exposure biomarkers with biomarkers of combined biological activity to PFAS mixtures in epidemiologic studies may be of great help [58]. Berg et al. (2017) [84] did not find an association between summed PFAAs exposure (sum of PFHpS, PFHxS, PFOA, PFOS, PFNA, PFDA and PFUnA) and TH outcome. Whereas, high exposure (4th quartile) to the summed POP (sumPFAAs + sumPCBs [sum of PCB 99,118, 138, 153, 163, 170, 180 and 187] + DDE + HCB + cis- and trans-nonachlor) was significantly positively associated with maternal TSH [84]. A study found no effect of the summed PFAAs exposure on maternal risk of hypothyroxinaemia (low free T4 without the compensatory rise in TSH) [85]. Furthermore, one study examined branched isomers of PFOS and found several significant findings, but not with the total PFOS [45].

The selected studies in this review investigated FT4I, FT4, FT3, TT4, TT3, T3RU and TSH in mothers and infants using different methods of chemoluminescent-, radio- or enzyme linked immunosorbents assays, with variable sensitivity (e.g. LOD (TSH) = 0.50 μU/mL [38] vs. LOD (TSH) = 0.01 μU/mL [41]). The radio immune sorbent assay seems to be the most reliable method for the quantification of thyroid hormones [86], but all methods showed good sensitivity, specificity, and accuracy. However, the use of immunosassay for hormone determination has been criticized. Animal studies suggested that the effect of PFOS to decrease FT4 level might be due to negative bias in analog techniques resulting from competitive displacement of FT4 and the labeled analog from serum and assay binding proteins in the presence of PFOS [61, 87]. Nevethrthless, such bias was not observed in human study and such different result between human and animal study might be related to the difference of PFAA level, protein binding to T4 (in rats: TTR, in human: TBG) and the interaction of proteins and PFAAs [88]. Based on these reported data, the use of different methods seems not to be of concern in measurements of human thyroid hormones.

Most studies examined TH at a single time point during pregnancy, impeding the capture of an effect on the thyroidal system during pregnancy since levels vary substantially during different pregnancy stages and stabilises approximately 6 weeks postpartum [34, 89]. However, longitudinal studies measure TH outcomes multiple times having a greater chance of witnessing an effect on the thyroid system [42, 43, 45].

TSH was examined in all studies except one [50], and T4 and T3 parameters were less consistently studied. The free TH reflects the biologically active hormones, which may give a better estimate of the exposure effect on the thyroid regulatory systems [90]. The FT4I is an estimate of circulating free T4 levels, which accounts for changes in T4 levels due to changes in thyroid binding protein levels and saturation, such as increases in TBG during pregnancy [91], being a better estimate, but only assessed in one single study [39]. Some of the studies only investigated a single TH parameter [41, 48, 50], however to understand the effect of PFAA exposure on the TH equilibrium at least T4 and TSH measurements are required.

The extent of adjustment for potential confounders varied largely between studies. General confounders were pre-pregnancy BMI, maternal age, socio-occupational status, parity, alcohol and smoking (Table 2). Eight studies excluded participants with thyroid diseases or related medication [38, 39, 41–44, 46, 48] and one study adjusted for these confounders [50]. However, few studies also took into account other thyroid parameters such as iodine status, TPOAb or thyroid binding capacity. Berg et al. demonstrated that several significant effect estimates lost significance after adjusting for thyroid binding capacity in the statistical analysis [43], suggesting an area that should be further studied.

FT4I was suggested as a more reliable FT4 estimate during pregnancy [39]. Weiss et al. found evidence that PFAAs might decrease T4 due to their affinity to thyroid hormone transport proteins using transthyretin (TTR) as carrier protein [55]. However, Preston et al. did not support this theory, after showing lower saturation of plasma binding proteins with higher PFAAs exposure [39]. The incoherent results may be due to the fact that experimental studies use TTR as carrier protein, although the major carrier protein in humans is TBG, which has lower PFAA affinity than TTR [92].

Three studies stratified for TPOAb-status and maternal TSH was associated with several PFAAs in the high TPOAb group [39, 42, 45]. However, the high TPOAb groups differed in sample size (e.g. 14 vs. 98 participants), gestational week (GW) of exposure sampling (median GW 9.6, GW 15–18 and multiple time points), TPO-Ab threshold (Webster et al. and Reardon et al.: > 9 IU/mL vs. Preston et al.: > 2 U/mL) and PFAA concentrations (lower in Reardon et al). Even though results went in different directions, it might indicate a more susceptible group among women with thyroid stress, whom may not be able to compensate for the PFAAs-induced thyroid affections [42]. If these women are highly exposed to PFAAs, which are strongly suspected to affect the thyroid gland, it could increase their risk of immune reactivity and affect the TH production necessary for a normal foetal development.

## Conclusion

In overall, most studies supported a positive association of maternal TSH and a possible negative association of maternal T4 and T3 upon PFAAs exposure. Infant TH might also be affected. The trimester data evaluation showed different direction of associations between PFOS and maternal TSH in different trimester. A positive association between TSH and PFOS was observed in longitudinal studies. The associations of PFAAs and TH in infant gender differed and were PFAA congeners dependent.

PFAAs exposure has a stronger influence on maternal than infant TH levels. Scientific evidence seems to favour an increase in maternal TSH and decrease T4 and T3 estimates upon PFAA exposure. The reviewed studies reported some associations between PFAAs and infant TH levels, although these data were less conclusive. The data did not indicate a clear trend in infant gender sensitivity. This review also showed a tendency of trimester dependency of maternal TSH levels, but more studies are needed longitually or during the 1st trimester with follow-up at birth with samples from mother and child (cord blood).

Future studies need a uniform method of examining the associations. We suggest future studies to focus on the 1st trimester with longitially followup in nulliparous women and include a broad range of PFAA congeners.

## Supplementary information

**Additional file 1.** Association of PFAAs with TSH and TH levels by infant sex.

## Data Availability

Not applicable. No datasets were generated or analyzed during the current study. All original manuscripts are available online at https://www.ncbi.nlm.nih.gov/pubmed/

## Abbreviations

BW: Birth weight
BL: Birth length
DDE: Dichlorodiphenyldichloroethylene
EtFOSAA: N-ethyl perfluorooctane sulfonamido acetic acid
6:2 FTS: 6:2 Fluorotelomer Sulfonate
HC: Head circumference
HCB: Hexachlorbenzen
HPG: Hypothalamic-Pituitary-Gonadal
HPT: Hypothalamic-Pituitary-thyroid
LOD: Limit of detection
LOQ: Limit of quantification
MeFOSAA: 2-(N-methylperfluorooctanesulfonamido) acetic acid
MESH: Medical Subject Headings
MoA: Mechanism of action
OCPs: Organochlorine pesticides
PCBs: Polychlorinated biphenyls
PFAAs: Perfluorinated-alkyl-acids
PFDA: Perfluorodecanoic acid
PFDoA: Perfluorododecanoic acid
PFHpA: Perfluoroheptanoic acid
PFHpS: Perfluoroheptane sulfonate
PFHxS: Perfluorohexane sulfonate
PFNA: Perfluorononanoic acid
PFOA: Perfluorooctanoate
PFOS: Perfluorooctane sulfonate
PFOSA: Perfluorooctane sulphonamide
PFPeA: Perfluoropentanoic acid
PFUnA: Perfluoroundecanoic acid
PFTrDA: Perfluorotridecanoic acid
PFTeDA: Perfluorotetradecanoic acid
POPs: Persistent organic pollutants
TBG: Thyroxin binding globulin
TH: Thyroid hormones
TSH: Thyroid stimulating hormone
T4: Thyroxine
T3: Triiodothyronine
T3RU: Free triiodothyronine resin uptake
TT4: Total Thyroxine
FT4: Free Thyroxine
FT4I: Free thyroxine index
TPO: Thyroid peroxidase
TPOab: Thyroid peroxidase antibodies
TTR: Transthyretin
UCB: Umbilical cord blood
SGA: Small for gestational age
