# Supplementary material for: Exposure to Perflouroalkyl acids and foetal and maternal thyroid status: a review
Source: Environ Health. 2020 Oct 13;19:107. doi: 10.1186/s12940-020-00647-1 (PMC7557068; doi:10.1186/s12940-020-00647-1)
Supplement: Supplementary file 1 — Additional file 1. Association of PFAAs with TSH and TH levels by infant sex. [file 12940_2020_647_MOESM1_ESM.docx]

# Supplementary Material

Exposure to Perflouroalkyl Acids and foetal and maternal thyroid status: a review.

*Sophie A. H. Boesen^1^, Manhai Long^1^, Maria Wielsøe^1^, Vicente Mustieles^2,3^, Mariana F. Fernandez ^2,3^, Eva C. Bonefeld-Jørgensen^1,4*^*

^1^ Centre for Arctic Health & Molecular Epidemiology, Department of Public Health Aarhus University, Denmark;

^2^ School of Medicine, Center of Biomedical Research, University of Granada, Spain.

^3^ Consortium for Biomedical Research in Epidemiology & Public Health (CIBERESP), Spain.

^4^ Greenland Centre for Health Research, University of Greenland, Nuuk, Greenland.

*^*^* **Corresponding Author:** Eva Cecilie Bonefeld-Jørgensen

The supplementary material contain four supplementary tables:

Suppl. Table 1a. Association of PFAAs and male infant TSH levels (adjusted results)

Suppl. Table 1b. Association of PFAAs and female infant TSH levels (adjusted results)

Suppl. Table 2a. Association of PFAAs and male infant T4 and T3 (adjusted results)

Suppl. Table 2b. Association of PFAAs and female infant T4 and T3 (adjusted results)

| Supplementary table 1: Association of PFAAs exposure and infant TSH stratified on infant sexSuppl. Table 1a: Association of PFAAs and male infant TSH levels (adjusted results) | | | | | | | | | |
| --- | --- | --- | --- | --- | --- | --- | --- | --- | --- |
| *Reference* | *Author Country Year* | *Effect estimate* | *PFHxS* | *PFOS* | *PFOA* | *PFNA* | *PFDA* | *PFUnA* | *PFDoA* |
| ***Maternal PFAAs concentrations obtained during 3^rd^ trimester*** | | | | | | | | | |
| *(38)* | Kato Japan 2016 | Linear regression: Adjusted β  (p value) | - | ↑: **0.205 (0.014)*** | ↓: -0.095 (0.277) | - | - | - | - |
| *(44)* | Wang Taiwan 2014 | No sex stratification performed | |  |  |  |  |  |  |
| *(46)* | Yang China 2016 | No sex stratification performed | |  |  |  |  |  |  |
| *(47)* | Xiao  Faroe Island  2019 | Percent change in thyroid hormone levels per doubling of PFAA concentrations (95% CI) | ↓: -2.5 (-28.8, 33.6) | ↑: **39.5 (0.4, 94.1)*** | ↑: 23.0 (-5.7, 60.6) | ↑: **60.6 (15.0, 124.6)*** | ↑: **37.0 (0.5, 86.8)*** | ↑: 15 (-8.3, 44.5) | ↑: 1.9 (-9.8, 15) |
| ***Cord blood serum/plasma PFAAs concentrations*** | | | | | | | | | |
| *(48)* | Dufour Belgium 2018 | Multivariate regression: Adjusted β (p value) | - | ↓: -0.042 (0.536) | ↓: -0.073 (0.316) | ↓: **-0.166* (0.018)** | - | - | - |
| *(49)* | Shah-Kulkarni Korea 2016 | Linear regression: Adjusted β (95% CI) | ↓: -1.15  (-3.05, 0.74) | ↑: 0.30  (-0.90, 1.52) | ↓: -0.31  (-2.00, 1.37) | ↓: 0.41  (-1.15, 1.98) | ↑: 0.24  (-1.99, 2.48) | ↓: -0.27  (-2.18, 1.63) | ↓: -0.67  (-3.35, 2.00) |
| *(51)* | Tsai Taiwan 2017 | Linear regression: Adjusted β (95% CI) | - | ↑: **0.333**  **(0.012, 0.678)*** | ↑: 0.001  (-0.289, 0.289) | ↑: 0.142  (-0.062, 0.346) | - | ↑: 0.047  (-0.099, 0.192) | - |
| *(52)* | Aimuzi  China  2019 | Sparse partial least squares (SPLS): Adjusted β (95% CI) | # | # | # | # | # | # | ↓: **-0.062**  **(-0.108, -0.017)*** |
| *TSH (thyroid stimulating hormone), ↓ decreasing, ↑ increasing, bold format and star* indicate significant results P<0.05, NS: non-significant, -: PFAAs not examined,* # *indicates association was not selected in SPLS model Perfluorooctane sulfonate (PFOS), Perfluorooctanoate (PFOA), Perfluorohexane sulfonate (PFHxS), Perfluorononanoic acid (PFNA), Perfluorodecanoic acid (PFDA), Perfluoroundecanoic acid (PFUnA), Perfluorododecanoic acid (PFDoA)* | | | | | | | | | |

| Suppl. Table 1b: Association of PFAAs and female infant TSH levels (adjusted results) | | | | | | | | | |
| --- | --- | --- | --- | --- | --- | --- | --- | --- | --- |
| *Reference* | *Author Country Year* | *Effect estimate* | *PFHxS* | *PFOS* | *PFOA* | *PFNA* | *PFDA* | *PFUnA* | *PFDoA* |
| ***Maternal PFAAs concentrations obtained during 3^rd^ trimester*** | | | |  |  |  |  |  |  |
| *(38)* | Kato Japan 2016 | Linear regression: Adjusted β  (p-value) | - | ↑: **0.173 (0.021)*** | ↑: 0.054 (0.465) | - | - | - | - |
| *(44)* | Wang Taiwan 2014 | No sex stratification performed | |  |  |  |  |  |  |
| *(46)* | Yang China 2016 | No sex stratification performed | |  |  |  |  |  |  |
| *(47)* | Xiao  Faroe Island  2019 | Percent change in thyroid hormone levels per doubling of PFAA concentrations (95% CI) | ↑: 24.4 (-6.6, 65.5) | ↑: 39.9 (-4.1, 104.2) | ↑: 23.1 (-5.1, 59.7) | ↑**: 46.7 (5.9, 103.2)*** | ↑: 26.0 (-7.0, 70.6) | ↑: 0.5 (-19.9, 26.1) | ↓: -1.4 (-11.9, 10.5) |
| ***Cord blood serum/plasma PFAAs concentrations*** | | | | | | | | | |
| *(48)* | Dufour Belgium 2018 | Multivariate regression: Adjusted β (p value) | - | ↓: -0.019 (0.883) | ↓: -0.112 (0.419) | ↓: - 0.074 (0.564) | - | - | - |
| *(49)* | Shah-Kulkarni Korea 2016 | Linear regression: Adjusted β (95% CI) | ↓: -0.84  (-3.24, 1.54) | ↓: -0.48  (-1.77, 0.81) | ↓: -1.05  (-3.22, 1.10) | ↓: **-1.69**  **(-3.31, -0.08**)* | ↓: -1.89  (-4.33, 0.54) | ↓: -0.76  (-2.82, 1.28) | ↓: -1.60  (-4.59, 1.39) |
| *(51)* | Tsai Taiwan 2017 | Linear regression: Adjusted β (95% CI) | - | ↑: 0.36  (-0.033, 0.753) | ↑: 0.177  (-0.122, 0.476) | ↓: -0.01  (-0.227, 0.208) | - | ↑: 0.048  (-0.092, 0.187) | - |
| *(52)* | Aimuzi  China  2019 | Sparse partial least squares (SPLS): Adjusted β (95% CI) | # | ↓: **-0.016**  **(-0.032, -0.002)*** | # | ↓: -0.015  (-0.031, 0.001) | ↓: -0.013  (-0.026, 0.01) | ↓: -0.018  (-0.033, 0.003) | # |
| *TSH (thyroid stimulating hormone), ↓ decreasing, ↑ increasing, bold format and star* indicate significant results P<0.05, -: PFAAs not examined, # indicates association was not selected in SPLS model Perfluorooctane sulfonate (PFOS), Perfluorooctanoate (PFOA), Perfluorohexane sulfonate (PFHxS), Perfluorononanoic acid (PFNA), Perfluorodecanoic acid (PFDA), Perfluoroundecanoic acid (PFUnA), Perfluorododecanoic acid (PFDoA)* | | | | | | | | | |

| Supplementary table 2: Association of PFAAs exposure and infant T4 and T3 estimates stratified on infant sex  Suppl. Table 2a: Association of PFAAs and male infant T4 and T3 (adjusted results) | | | | | | | | | | | | | |
| --- | --- | --- | --- | --- | --- | --- | --- | --- | --- | --- | --- | --- | --- |
| *Reference* | *Author Country Year* | *Effect estimate* | *TH* | *PFHxS* | *PFOS* | *PFOA* | *PFNA* | | *PFDA* | | *PFUnA* | | *PFDoA* |
| ***Maternal PFAAs concentrations obtained during 1^st^ Trimester*** | | | | | | | | | | | | | |
| (39) | Preston USA 2018 | Difference in male infant TH levels per quartile (Q2-4 vs. Q1) maternal PFAAs concentrations (95% CI) | TT4 | ↓: Q2: -1.17  (-2.69, 0.35) | ↓: Q2: **-1.56**  **(-3.04, -0.08)** | ↓: Q2: -0.75  (-2.30, 0.81) | ↓: Q2: -0.75  (-2.32, 0.82) | | - | | - | | - |
|  |  |  |  | ↓: Q3: **-1.63**  **(-3.08, -0.19)** | ↓: Q3: **-1.70**  **(-3.28, -0.12)** | ↓:Q3: -1.32 (-2.85, 0.21) | ↓: Q3: -1.00  (-2.40, 0.40) | | - | | - | | - |
|  |  |  |  | ↓: Q4: **-2.51**  **(-3.99, -1.04)** | ↓: Q4: **-2.20**  **(-3.74, -0.66)** | ↓: Q4: **-1.72**  **(-3.36, -0.08)** | ↓: Q4: -1.14  (-2.60, 0.33) | | - | | - | | - |
| ***Maternal PFAAs concentrations obtained during 3^rd^ Trimester*** | | | | | | | | | | | | | |
| *(38)* | Kato Japan 2016 | Linear regression: Adjusted β  (p-value) | FT4 | - | ↓: -0.037 (0.670) | ↑: 0.023  (0.795) | - | - | | - | | - | |
| *(44)* | Wang Taiwan 2014 | No sex stratification performed | |  |  |  |  |  | |  | |  | |
| *(46)* | Yang China 2016 | No sex stratification performed | |  |  |  |  |  | |  | |  | |
| *(47)* | Xiao  Faroe Island  2019 | Percent change in thyroid hormone levels per doubling of PFAA concentrations (95% CI) | FT4I | ↑: 1.4 (-8.0, 11.7) | ↑: 2.1 (-7.7, 13.0) | ↓: -5.2 (-12.5, 2.7) | ↑: 9.3 (-1.6, 21.4) | | ↑: **11.5 (1.5, 22.6)*** | | ↑: 4.3 (-2.7, 11.7) | | ↓: -2.3 (-5.8, 1.4) |
|  |  |  | FT3 | ↓: -1.0 (-10.7, 9.7) | ↑: 2.2 (-8.4, 14.1) | ↓: -2.7 (-10.9, 6.3) | ↑: 5.4 (-5.8, 18.1) | | ↑: 4.6 (-5.5, 15.8) | | ↑: 3.5 (-4, 11.4) | | ↓: -0.8 (-4.6, 3.2) |
|  |  |  | FT4 | ↑: 18.1 (-6.1, 48.4) | ↑: 8.3 (-15.1, 38.3) | ↑: 0.8 (-17.4, 22.9) | ↑: 5.8 (-18.0, 36.5) | | ↑: 7.5 (-14.7, 35.3) | | ↓: -2.1 (-17.1, 15.7) | | ↑: 0.8 (-7.8, 10.1) |
|  |  |  | TT4 | ↓: -2.6 (-12.0, 7.8) | ↓: -2.3 (-12.2, 8.8) | ↓: -5.2 (-12.8, 3.3) | ↑: 4.0 (-7.0, 16.2) | | ↑: 7.9 (-2.4, 19.2) | | ↑: 1.3 (-5.8, 9) | | ↓: -3.0 (-6.6, 0.9) |
|  |  |  | T3RU | ↑: 3.9 (-1.0, 9.0) | ↑: 3.4 (-1.8, 8.9) | ↓: -0.1 (-4.2, 4.1) | ↑: 4.2 (-1.2, 9.9) | | ↑: 2.9 (-2.0, 8.0) | | ↑: 2.3 (-1.1, 6) | | ↑: 0.3 (-1.6, 2.1) |
| ***Cord blood serum/plasma PFAAs concentrations*** | | | |  |  |  |  | |  | |  | |  |
| *(49)* | Shah-Kulkarni Korea 2016 | Linear regression: Adjusted β (95% CI) | TT3 | ↑: 0.02 (-3.06, 3.10) | ↓: -0.34 (-2.31, 1.61) | ↓: -1.36 (-4.09, 1.35) | ↓: -1.03 (-3.56, 1.49) | | ↑: 1.02 (-2.58, 4.62) | | ↑: 0.40 (-2.67, 3.49) | | ↑: 2.49 (-1.81, 6.81) |
|  |  |  | TT4 | ↓: -0.12 (-0.52, 0.28) | ↑: 0.05 (-0.19, 0.31) | ↓: -0.12 (-0.47, 0.23) | ↓: -0.09 (-0.42, 0.23) | | ↑: 0.05 (-0.41, 0.53) | | ↓: -0.01 (-0.40, 0.39) | | ↓: -0.08 (-0.65, 0.47) |
| *(50)* | De Cock Netherland 2014 | Linear regression coefficients (95% CI): Change across quartiles (Q1 reference) | TT4 | - | ↓: Q2: -7.9 (-31.56, 15.74) | ↑: Q2: 7.9 (-18.04, 33.92) | - | | - | | - | | - |
|  |  |  |  |  | ↓: Q3: -16.5 (-40.32, 7.34) | ↓: Q3: -2.1 (-20.94, 16.78) |  | |  | |  | |  |
|  |  |  |  |  | ↓: Q4: -9.6 (-32.57, 13.31) | ↑: Q4: 6.2 (-16.08, 28.50) |  | |  | |  | |  |
| *(51)* | Tsai Taiwan 2017 | Linear regression: Adjusted β (95% CI) | TT4 | - | ↓: **-0.667**  **(-1.283, -0.05)*** | ↓: -0.082  (-0.643, 0.48) | ↓: -0.128  (-0.41, 0.154) | | - | | ↓: -0.082  (-0.485, 0.321) | | - |
|  |  |  | TT3 | - | ↓: -0.035  (-0.16, 0.091) | ↑: 0.017  (-0.092, 0.126) | ↑: 0.05  (-0.027, 0.127) | | - | | ↑: 0.047  (-0.101, 0.007) | | - |
| *(52)* | Aimuzi  China  2019 | Sparse partial least squares (SPLS): Adjusted β (95% CI) | FT4 | ↑: 0.024  (-0.024, 0.067) | ↑: 0.023  (-0.035, 0.057) | **↑: 0.062**  **(0.024, 0.138)*** | **↑: 0.04**  **(0.006, 0.081)*** | | **↑: 0.043**  **(0.016, 0.078)*** | | ↑: 0.033  (-0.007, 0.069) | | **↑: 0.54**  **(0.019, 0.119)*** |
|  |  |  | FT3 | # | ↑: 0.034  (-0.069, 0.185) | ↓: -0.068  (-0.151, 0.015) | ↑: 0.075  (-0.07, 0.216) | | # | | ↑: 0.107  (-0.069, 0.234) | | ↓: -0.251  (-0.361, 0.144) |
| *TT3 (total triiodothyronine), FT3 (total triiodothyronine), TT4 (total thyroxine), FT4 (free thyroxine), FT4I (free thyroxine index), ↓ decreasing, ↑ increasing, bold format and star* indicate significant results P<0.05, NS: non-significant, -: PFAAs not examined. Q1 (1st quartile), Q2 (2nd quartile), Q3 (3rd quartile), Q4 (4th quartile), IQR (interquartile range), # indicates association was not selected in SPLS model.*  *PFAAs include Perfluorooctane sulfonate (PFOS), Perfluorooctanoate (PFOA), Perfluorohexane sulfonate (PFHxS), Perfluorononanoic acid (PFNA), Perfluorodecanoic acid (PFDA), Perfluoroundecanoic acid (PFUnA), Perfluorododecanoic acid (PFDoA).* | | | | | | | | | | | | | |

| Suppl. table 2b: Association of PFAAs and female infant T4 and T3 (adjusted results) | | | | | | | | | | | | | | | | |
| --- | --- | --- | --- | --- | --- | --- | --- | --- | --- | --- | --- | --- | --- | --- | --- | --- |
| *Reference* | *Author Country Year* | *Effect estimate* | *TH* | *PFHxS* | | *PFOS* | | *PFOA* | | *PFNA* | | *PFDA* | | *PFUnA* | | *PFDoA* |
| ***Maternal PFAAs concentrations obtained during 1^st^ Trimester*** | | | | | | | | | | | | | | | | |
| (39) | Preston USA 2018 | Difference in female infant TH levels per quartile (Q2-4 vs. Q1) maternal PFAAS concentrations (95% CI) | TT4 | ↓: Q2: -0.51  (-1.91, 0.90) | | ↑: Q2 0.28  (-1.12, 1.67) | | ↓: Q2: -0.21  (-1.62, 1.19) | | ↓: Q2: -0.30  (-1.86, 1.26) | | - | | - | | - |
|  |  |  |  | ↓: Q3: -0.42  (-1.78, 0.94) | | ↑: Q3 0.68  (-0.71, 2.07) | | ↑: Q3 0.53  (-0.93, 1.99) | | ↓: Q3: -0.31  (-1.55, 0.92) | | - | | - | | - |
|  |  |  |  | ↑: Q4: 0.40  (-0.98, 1.79) | | ↓: Q4: -0.09  (-1.52, 1.33) | | ↓: Q4: -0.67  (-2.13, 0.78) | | ↑: Q4 0.01  (-1.48, 1.50) | | - | | - | | - |
| ***Maternal PFAAs concentrations obtained during 3^rd^ Trimester*** | | | | | | | | | | | | | | | | |
| (38) | Kato Japan 2016 | Linear regression: Adjusted β  (p value) | FT4 | - | | ↓: -0.044  (0.567) | | ↑: -0.017  (0.819) | | - | | - | | - | | - |
| (44) | Wang Taiwan 2014 | No sex stratification performed | | |  | |  | |  | |  | |  | |  | |
| (46) | Yang China 2016 | No sex stratification performed | | |  | |  | |  | |  | |  | |  | |
| (47) | Xiao  Faroe Island  2019 | Percent change in thyroid hormone levels per doubling of PFAA concentrations (95% CI) | FT4I | ↓: -0.6 (-9.0, 27.1) | | ↑: **13.2 (0.9, 27.1)*** | | ↑: **10.7 (2.4, 19.6)*** | | ↑: 7.1 (-3.2, 18.6) | | ↑: 5.5 (-3.7, 15.7) | | ↑: 2.0 (-4.8, 9.3) | | ↑: 0.9 (-2.6, 4.4) |
|  |  |  | FT3 | -↓: 3.5 (-12.2, 5.9) | | ↓: -4.8 (-16.1, 8.0) | | ↑: 3.5 (-5.1, 12.7) | | ↓: -3.1 (-13.2, 8.1) | | ↓: -2.2 (-11.5, 8.2) | | ↑: 1 (-6.2, 8.8) | | ↓: -2.1 (-5.5, 1.6) |
|  |  |  | FT4 | ↑: 11.2 (-9.7, 36.8) | | ↑: 26.4 (-4.5, 67.2) | | ↑: 2.9 (-15.1, 25.0) | | ↑: 6.2 (-17.1, 36.2) | | ↑: 7.7 (-14.1, 34.9) | | ↑: 1.3 (-14.2, 19.7) | | ↑: 2.4 (-5.7, 11.2) |
|  |  |  | TT4 | ↓: -2.4 (-11.0, 7.0) | | ↑: 9.5 (-3.1, 23.7) | | ↑: **9.1 (0.5, 18.5)*** | | ↑: 4.3 (-6.5, 16.2) | | ↑: 3.3 (-6.4, 13.9) | | ↓: -0.1 (-7.1, 7.5) | | ↓: -0.3 (-3.8, 3.3) |
|  |  |  | T3RU | ↑: 1.9 (-2.4, 6.5) | | ↑: 3.8 (-2.2, 10.1) | | ↑: 1.3 (-2.8, 5.4) | | ↑: 3.0 (-2.1, 8.5) | | ↑: 2.6 (-2.1, 7.6) | | ↑: 2.4 (-1.1, 6.1) | | ↑: 1.2 (-0.5, 3) |
| ***Cord blood serum/plasma PFAAs concentrations*** | | | | | | | | | | | | | | | | |
| (49) | Shah-Kulkarni Korea 2016 | Linear regression: Adjusted β (95% CI) | TT3 | ↑: **4.28**  **(0.39, 8.17)*** | | ↑: 1.67  (−0.44, 3.79) | | ↑: 2.04  (−1.51, 5.61) | | ↑: 1.32  (−1.36, 4.02) | | ↑: 3.93  (−0.07, 7.93) | | ↑: 2.39  (−0.98, 5.76) | | ↑: 2.03  (−2.91, 6.98) |
|  |  |  | TT4 | ↑: 0.19  (−0.25, 0.64) | | ↑: 0.23  (−0.01, 0.47) | | ↑: 0.16  (−0.24, 0.57) | | ↑: 0.17  (−0.13, 0.47) | | ↑: 0.23  (−0.22, 0.69) | | ↓: −0.01 (−0.39, 0.38) | | ↓: −0.03  (−0.59, 0.53) |
| (50) | De Cock Netherland 2014 | Linear regression coefficients (95% CI): Change across quartiles (Q1 reference) | TT4 |  | | ↓: Q2: -1.3  (-30.45, 27.94) | | ↓: Q2: -5.9  (-26.075, 14.94) | |  | |  | |  | |  |
|  |  |  |  | - | | ↑: Q3: 4.5  (-25.95, 34.92) | | ↑: Q3: 11.8  (-19.08, 42.72) | | - | | - | | - | | - |
|  |  |  |  |  | | ↑: Q4: 15.9  (-10.67, 42.40) | | **↑: Q4: 38.6 (13.34, 63.83)*** | |  | |  | |  | |  |
| (51) | Tsai Taiwan 2017 | Linear regression: Adjusted β (95% CI) | TT4 | - | | ↑: 0.033  (-0.707, 0.773) | | ↑: 0.084  (-0.483, 0.651) | | ↓: -0.02  (-0.282, 0.241) | | - | | ↑: 0.253  (-0.147, 0.653) | | - |
|  |  |  | TT3 | - | | ↑: 0.114 (-0.058, 0.286) | | ↑: 0.03 (-0.104, 0.164) | | ↓: -0.01 (-0.072, 0.052) | | - | | ↑: 0.041  (-0.055, 0.137) | | - |
| (52) | Aimuzi  China  2019 | Sparse partial least squares (SPLS): Adjusted β (95% CI) | FT4 | # | | # | | # | | # | | # | | # | | **↑: 0.174**  **(0.019, 0.331)*** |
|  |  |  | FT3 | # | | # | | # | | # | | # | | # | | **↓: -0.124**  **(-0.185, -0.056)*** |
| *TT3 (total triiodothyronine), FT3 (total triiodothyronine), TT4 (total thyroxine), FT4 (free thyroxine), FT4I (free thyroxine index), ↓ decreasing, ↑ increasing, bold format and star*indicate significant results P<0.05, NS: non-significant, -: PFAAs not examined. Q1 (1^st^ quartile), Q2 (2^nd^ quartile), Q3 (3^rd^ quartile), Q4 (4^th^ quartile), IQR (interquartile range),* # *indicates association was not selected in SPLS model.* | | | | | | | | | | | | | | | | |
| *PFAAs include Perfluorooctane sulfonate (PFOS), Perfluorooctanoate (PFOA), Perfluorohexane sulfonate (PFHxS), Perfluorononanoic acid (PFNA), Perfluorodecanoic acid (PFDA), Perfluoroundecanoic acid (PFUnA), Perfluorododecanoic acid (PFDoA).* | | | | | | | | | | | | | | | | |
